# Inhibition of autophagic flux by cyclometalated iridium(iii) complexes through anion transportation†
†Electronic supplementary information (ESI) available: Syntheses and characterization data, anion transport assays, cell lines and culture conditions, cell viability assays, transmission electron microscopy, western blotting, flow cytometry analysis. CCDC 1814116 and 1814117. For ESI and crystallographic data in CIF or other electronic format see DOI: 10.1039/c8sc04520h


**DOI:** 10.1039/c8sc04520h

**Published:** 2019-01-31

**Authors:** Mu-He Chen, Yue Zheng, Xiong-Jie Cai, Hang Zhang, Fang-Xin Wang, Cai-Ping Tan, Wen-Hua Chen, Liang-Nian Ji, Zong-Wan Mao

**Affiliations:** a MOE Key Laboratory of Bioinorganic and Synthetic Chemistry , School of Chemistry , Sun Yat-Sen University , Guangzhou 510275 , P. R. China . Email: tancaip@mail.sysu.edu.cn ; Email: cesmzw@mail.sysu.edu.cn; b Guangdong Provincial Key Laboratory of New Drug Screening , School of Pharmaceutical Sciences , Southern Medical University , Guangzhou 510515 , P. R. China . Email: whchen@smu.edu.cn

## Abstract

Synthetic anion transporters that can interfere with the intracellular pH homeostasis are gaining increasing attention for tumor therapy, however, the biological mechanism of anion transporters remains to be explored. In this work, two phosphorescent cyclometalated Ir(iii) complexes containing 2-phenylpyridine (ppy) as the cyclometalated ligand, and 2,2′-biimidazole (H_2_biim, **Ir1**) or 2-(1*H*-imidazol-2-yl)pyridine (Hpyim, **Ir2**) as the ancillary ligands have been synthesized and characterized. Due to the protonation and deprotonation process of the N–H groups on H_2_biim and Hpyim, **Ir1** and **Ir2** display pH-dependent phosphorescence and can specifically image lysosomes. Both **Ir1** and **Ir2** can act as anion transporters mainly through the anion exchange mechanism with higher potency observed for **Ir1**. Mechanism investigation shows that **Ir1** and **Ir2** can induce caspase-independent cell death through reactive oxygen species (ROS) elevation. As **Ir1** and **Ir2** can alkalinize lysosomes through anion disturbance, they can inhibit autophagic flux. Our work provides a novel anticancer mechanism of metal complexes, which gives insights into the innovative structure-based design of new metallo-anticancer agents.

## Abstract 2

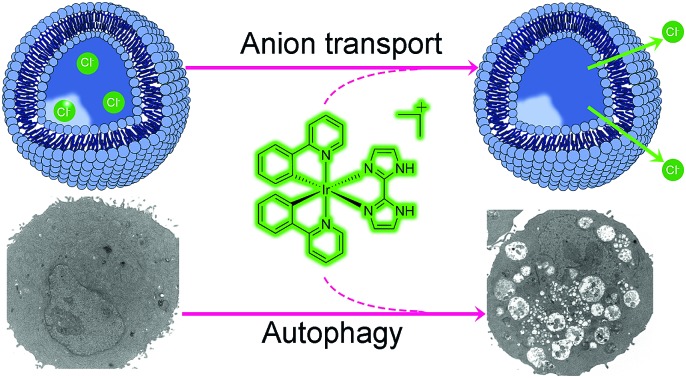
We report two phosphorescent cyclometalated iridium(iii) complexes that can inhibit autophagic flux through anion transportation.

## Introduction

Autophagy is an important process for cells to recycle waste.^1^ When autophagy is initiated, various damaged organelles and proteins are encapsulated into the bilayer membrane vesicles, forming autophagosomes. Subsequently, autophagosomes fuse with lysosomes to form autolysosomes. Hydrolytic enzymes in lysosomes degrade the contents of autolysosomes into nutrients that can be re-used by the cell. The relationship between autophagy and cancer is not yet fully understood. Autophagy is described as a double-edged sword in oncology.^2^,^3^ The prevailing view is that autophagy is primarily cytoprotective.^4^ However, when autophagy is extensive and prolonged, cell death may be induced.^4^–^6^

Cancer cells have many different hallmarks compared with normal cells.^7^ Dysregulated pH is gradually being considered as one of the hallmarks of cancer.^8^ Compared with normal cells, cancer cells have a higher intracellular pH and a lower extracellular pH to facilitate tumor proliferation and survival.^9^ Recently, researchers have begun to reverse the pH gradient of cells by various means and use this method as a new anticancer strategy.^10^ Anions are important for cells to maintain their pH homeostasis.^11^ Therefore, synthetic small-molecule anion transporters that can promote the transport of anions through lipophilic membranes and perturb the pH homeostasis are gaining attention as promising anticancer agents.^11^–^14^

Organometallic anticancer iridium complexes have drawn the attention of researchers over the past decade because of their different anticancer mechanisms from cisplatin and their potential to overcome cisplatin resistance and side effects.^15^–^18^ In particular, phosphorescent cyclometalated iridium(iii) complexes have been widely applied in bioimaging and biosensing due to their high photostability, large Stokes' shifts, relatively long emission life-times and environment-sensitive luminescence properties.^17^ Moreover, the long-lived phosphorescence of the iridium complexes allows for time-gated detection of the signal that can eliminate the short-lived autofluorescence or scattering to produce images with lower background noise.^19^ In the past few years, cyclometalated iridium(iii) complexes have already found a variety of biological applications, such as metallodrugs, biomolecular probes, and living cell imaging agents.^20^–^24^ It has been demonstrated that they can act as theranostic anticancer agents by integrating the imaging and therapeutic capabilities into one single molecule.^25^–^28^

Synthetic small-molecule anion transporters reported to date are mainly organic small molecules, such as prodigiosin analogues,^29^–^31^ squaramide derivatives,^32^–^34^ tris-(2-aminoethyl)amine (tren)-based compounds^35^,^36^ and calix[4]pyrrole derivatives.^12^,^37^–^39^ To the best of our knowledge, no metal complexes have been reported as anion transporters. In addition, our understanding of the effects of anion transporters on cells is still at a relatively early stage. New types of easy-to-make anion transporters are needed to better understand the mechanism of action of anion transporters at cellular levels. Metal complexes have some advantages to be developed as the anion transporters. For example, Ir complexes have diverse coordination structures, and their lipophilicity can be easily tuned. They have high capability to penetrate cancer cell membranes and can image the biological processes at subcellular levels.

**Fig. 1:**
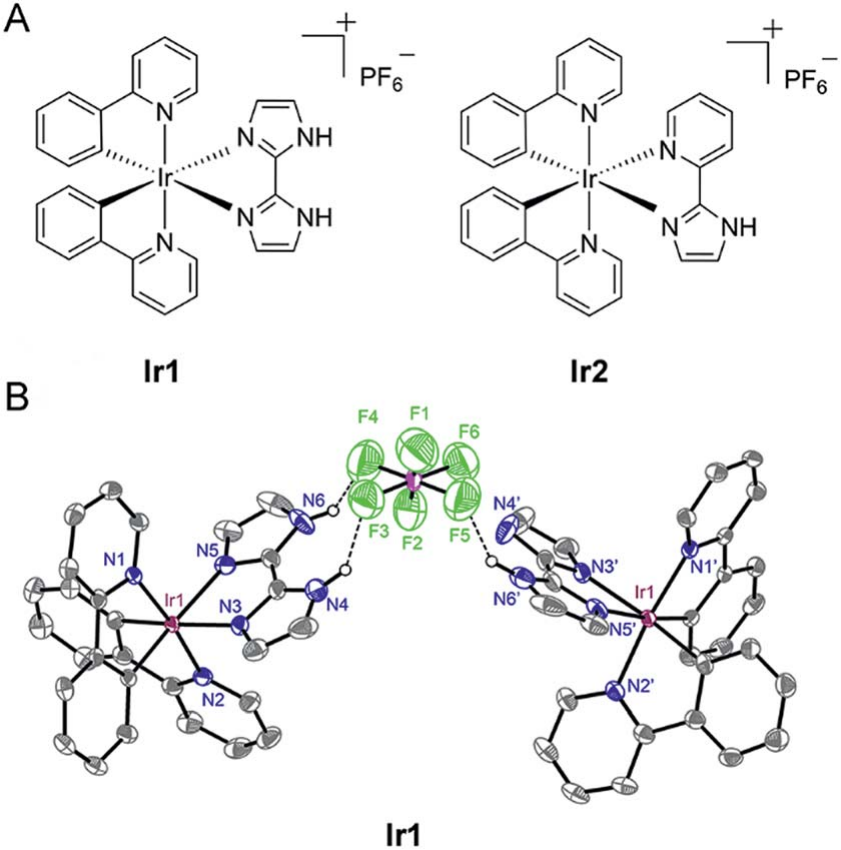
(A) Schematic drawing of the structures of **Ir1** and **Ir2**. (B) X-ray structure of **Ir1** is shown in thermal ellipsoids at the 50% probability level. For the sake of clarity, hydrogen atoms are omitted except for the hydrogen atoms that are attached to the nitrogen atoms.

In the present study, we designed two cyclometalated Ir(iii) complexes employing 2,2′-biimidazole (H_2_biim) (**Ir1**) or 2-(1*H*-imidazol-2-yl) pyridine (Hpyim) (**Ir2**) as the ancillary ligands and 2-phenylpyridine (ppy) as the cyclometalated ligand (Fig. 1A). H_2_biim in **Ir1** can act as the anion binding moiety as it contains two N–H groups. **Ir2** containing Hpyim with only one N–H group as the protonation and deprotonation site is studied for comparison purposes. Mechanism investigations show that **Ir1** can promote cell death by elevation of reactive oxygen species (ROS) levels. As expected, **Ir1** can function as an anion transporter and increase the lysosomal pH, leading to autophagic flux inhibition. To our knowledge, this is the first time that metal complexes are reported as anion transporters and their anticancer mechanisms are linked to anion transportation. Our findings provide new insights into the mechanism investigations of metallo-anticancer drugs.

## Results and discussion

### Synthesis and photophysical properties

**Ir1** (ref. 40) and **Ir2** were obtained by the direct reaction of H_2_biim or Hpyim with the dimeric Ir(iii) precursor [Ir(ppy)_2_Cl]_2_ in CH_2_Cl_2_/CH_3_OH (2 : 1, v/v). The crude products were purified by column chromatography on silica gel. Both complexes were characterized by ESI-MS, ^1^H NMR, ^13^C NMR, elemental analysis and X-ray crystallography (Fig. 1B and S1–S5†). The purity of **Ir1** and **Ir2** is >97% as measured by HPLC. **Ir1** and **Ir2** are stable in human plasma for 72 h from the HPLC-MS traces (Fig. S6 and S7†). In **Ir1**, two N–H…F hydrogen bonds are formed inside a positive and negative ion pair, and in addition, an N–H…F hydrogen bond is found between the same anion and another neutral Ir(iii) moiety lacking one proton. Such strong hydrogen-bonding interactions of **Ir1** with anions indicate the potential of **Ir1** as an anion transporter.

The UV/Vis absorption spectra of **Ir1** and **Ir2** were recorded in phosphate buffer saline (PBS), CH_2_Cl_2_ and CH_3_CN at room temperature (Fig. S8†). The intense energy absorption bands (250–350 nm) of **Ir1** and **Ir2** can be attributed to the spin-allowed ligand-centered transitions (^1^LC). The low energy absorption bands at 350–400 nm are assigned to a combination of spin-allowed metal-to-ligand charge transfer (^1^MLCT) and ligand-to-ligand charge transfer (^1^LLCT) processes. The lowest absorption tails (400–450 nm) can be assigned to spin-forbidden ^3^MLCT and ^3^LLCT transitions.^41^**Ir1** and **Ir2** emit green (500–550 nm) light in PBS, CH_2_Cl_2_ and CH_3_CN at room temperature upon excitation at 405 nm (Fig. S9†). Three excited states (^3^LC, ^3^MLCT and ^3^LLCT) possibly contribute to the emission of **Ir1** and **Ir2**.^41^ Detailed photophysical characteristics are summarized in Table S3.† The luminescence lifetimes of **Ir1** and **Ir2** range from 27.44 to 132.58 ns and their quantum yields fall between 2% and 13%. Both the emission intensities and lifetimes are solvent-dependent. **Ir1** and **Ir2** have higher quantum yields in CH_3_CN than in PBS and CH_2_Cl_2_. Besides, **Ir1** and **Ir2** show higher lifetimes in PBS or CH_2_Cl_2_ than in CH_3_CN.

### Protonation/deprotonation processes

**Fig. 2:**
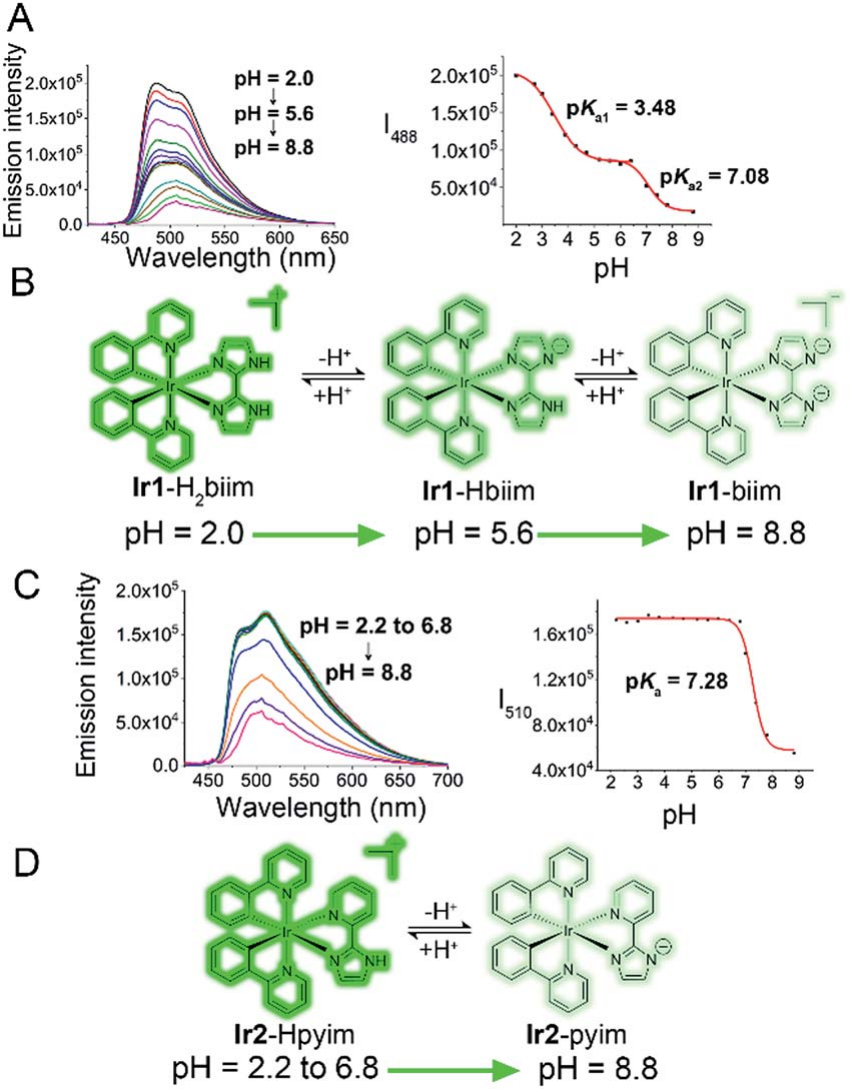
(A and C) pH-Dependent emission spectra and plots of the emission intensity of **Ir1** ((A) *λ*_em_ = 488 nm) and **Ir2** ((C) *λ*_em_ 510 nm) (20 μM, *λ*_ex_ = 405 nm) *versus* pH. Arrows indicate that the phosphorescence intensity decreases as the pH increases. (B and D) Protonation/deprotonation process of **Ir1** (B) and **Ir2** (D).

Under physiological conditions, compounds with suitable p*K*_a_ values can go through reversible protonation/deprotonation processes, which can change their electronic states and photophysical properties.^42^ The acidity of free H_2_biim and Hpyim is low (p*K*_a_ > 11).^43^,^44^ However, it has been reported that the acidity of imidazole derivatives increases upon coordination with metal ions due to the stronger stability of the ligand anion, so imidazoles in metal complexes are more susceptible to deprotonation.^43^ Both UV/Vis absorption spectra and emission spectra of **Ir1** and **Ir2** exhibit pH-dependent properties (Fig. 2A and C, S10†). The effect of pH on the absorption spectra of the complexes is relatively small. **Ir1** and **Ir2** display higher absorption peaks in a more alkaline environment (Fig. S10†). The effect of pH on the emission spectra of **Ir1** and **Ir2** is more significant, and results also show that the protonation/deprotonation processes of **Ir1** and **Ir2** are different (Fig. 2B and D). Both **Ir1** and **Ir2** are in the turn-off state and emit weak phosphorescence at neutral and basic pH (pH ≥ 7.4). When the pH decreases to about 2.0, the emission intensity increases about 12- and 3-fold for **Ir1** and **Ir2**, respectively. **Ir1** with the H_2_biim ligand has two protonated/deprotonated sites with two p*K*_a_ values being 3.48 and 7.08. It can be inferred that **Ir1** exists mainly in the **Ir1**-Hbiim or **Ir1**-biim form in the cytoplasm (pH ≈ 7.4) and in the **Ir1**-H_2_biim or **Ir1**-Hbiim form in acidic organelles, *e.g.*, lysosomes and endosomes (pH 4.7–6.3).^45^**Ir2** has only one protonated/deprotonated site (p*K*_a_ = 7.28), so **Ir2** exists mainly in the **Ir2**–pyim form in the cytoplasm and in the **Ir2**–Hpyim form in acidic organelles. The complexes will become neutral or charged when the protons lose. The off-on effect makes **Ir1** and **Ir2** suitable for imaging intracellular low pH environments, such as lysosomes and endosomes.

### Anion transport capability

Next, we tested the anion transport ability of **Ir1** and **Ir2** by a conventional method.^46^ Vesicles made of egg-yolk l-α-phosphatidylcholine (EYPC) containing 500 mM NaCl and 5 mM citric-phosphate buffer (pH 7.2) were suspended in isotonic and chloride-free 500 mM NaNO_3_ solutions with citric-phosphate buffer (pH 7.2). After adding the DMSO solution of **Ir1** and **Ir2**, a chloride ion selective electrode was used to monitor the chloride efflux in the solution. The vesicles were lysed by adding 5 wt% Triton X-100 at 300 s and the electrode reading was taken as 100% chloride release.

**Fig. 3:**
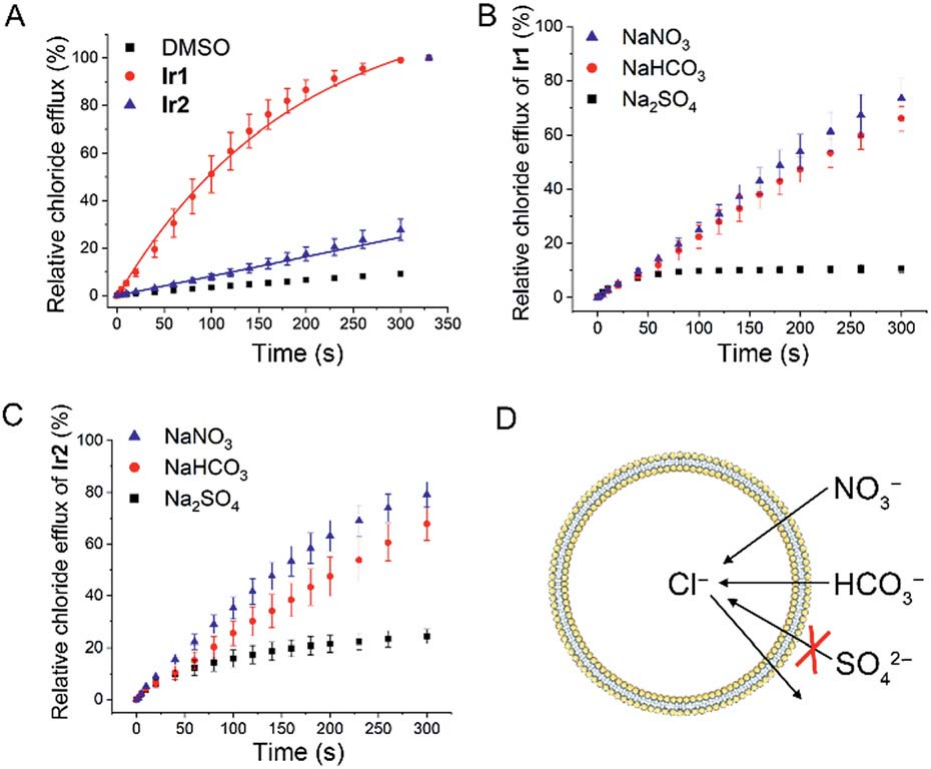
(A) Chloride/nitrate transport mediated by **Ir1** and **Ir2** (0.25 mol% with respect to lipid). Vesicles loaded with 500 mM NaCl and 5 mM citric-phosphate buffer (pH 7.2) dispersed in a 500 mM NaNO_3_ solution with 5 mM phosphate salts (pH 7.2). DMSO was used as a control. The initial rate of chloride transport (*k*_ini_) was calculated through asymptotic or linear fit. (B and C) Anion transport selectivity of **Ir1** ((B) 0.125 mol% with respect to lipid) and **Ir2** ((C) 3 mol% with respect to lipid). Vesicles loaded with 500 mM NaCl and 5 mM citric-phosphate buffer (pH 7.2) immersed in a 500 mM NaNO_3_, 500 mM NaHCO_3_ or 250 mM Na_2_SO_4_ in 5 mM citric-phosphate buffer (pH 7.2). (D) Schematic diagram showing the process of Cl^–^/HCO_3_^–^ exchange and Cl^–^/NO_3_^–^ exchange. Sulfate anions are difficult to be transported across the lipid bilayer due to their high hydrophilicity.

As shown in Fig. 3A and S11,† **Ir1** transports about 86% of the chloride anions in 200 s, whereas **Ir2** transports only about 17% under the same conditions. By comparing the initial rate of chloride transport (*k*_ini_), we know that **Ir1** (*k*_ini_ = 0.68% s^–1^) has a much higher chloride transport capacity than **Ir2** (*k*_ini_ = 0.08% s^–1^).

To further confirm this conclusion, we performed pH-dependent and concentration-dependent anion transport experiments (Fig. S12–S23†). By Hill analysis, we obtained the EC_50_ values of **Ir1** and **Ir2** under various conditions (Table S4†). The EC_50_ values that refer to the concentration of a carrier required to release 50% of chloride anions after the same time period can be used to compare the transport ability of different compounds under different conditions.

When the pH inside and outside the vesicles increases from 4.0 to 7.2, the transport activities of both **Ir1** and **Ir2** increase. **Ir1** displays higher transport activity than **Ir2**, and the EC_50_ value of **Ir1** can reach 0.038 mol% (with respect to lipid). Moreover, the transport activity of **Ir1** and **Ir2** is mainly affected by the pH outside the vesicle, which implies that the deprotonation process has a profound effect on anion transport activity. Similar transport activity measurements were carried out by suspending the vesicles in NaHCO_3_ or Na_2_SO_4_ solutions (Fig. 3B and C). It can be seen that **Ir1** and **Ir2** can carry out Cl^–^/HCO_3_^–^ transport in addition to Cl^–^/NO_3_^–^ transport (Fig. 3D). Only a low level of Cl^–^/SO_4_^2–^ can be transported by **Ir1** and **Ir2**. The strong hydrophilicity of sulfate anions makes themselves difficult to be transported across the lipid bilayer, so only a very small degree of chloride efflux can be observed when sulfate is the only anion in the external solution. The results imply that **Ir1** and **Ir2** transport anions primarily through an anion exchange mechanism.

Different metal cations (Li^+^, Na^+^, K^+^, Rb^+^ and Cs^+^) do not cause significant difference in the anion transport activity of **Ir1** and **Ir2**, so metal cations are not the main determinants of anion transport (Fig. S24†). To investigate whether **Ir1** and **Ir2** transport anions through a mechanism of ion channels or carriers, we added cholesterol to the liposomes (EYPC : cholesterol = 7 : 3 in molar ratio) and then tested its effect on the anion transport activity of **Ir1** and **Ir2**. Cholesterol is thought to increase the viscosity of lipid membranes and reduce the diffusion within the lipid bilayers. Cholesterol can greatly influence mobile carriers, but has little effect on ion channels.^32^ The addition of cholesterol significantly reduces the anion transport activity of **Ir1** and **Ir2**, indicating that **Ir1** and **Ir2** do not transport the anion through a channel mechanism (Fig. S25†). Calcein leakage assays show that **Ir1** and **Ir2** do not cause membrane rupture that can lead to non-specific chloride excretion (Fig. S26†).

To test whether the complexes have anion transport activity in cells, we chose a chloride-quenching fluorescent indicator *N*-(ethoxycarbonylmethyl)-6-methoxyquinolinium bromide (MQAE) to detect the concentration of intracellular chloride ions (Fig. S27†).^12^,^34^,^47^ The fluorescence of MQAE is significantly reduced, which indicates that the complexes can induce chloride ion influx in cells. Meanwhile, the intracellular chloride concentrations of the control group are not altered. These results indicate that the complexes possess anion transport activity in cells.

### *In vitro* cytotoxicity

We tested the octanol–water partition coefficient (log *P*_o/w_) of **Ir1** and **Ir2** and their cytotoxicity towards different cancer cell lines, including human cervical cancer (HeLa), human lung adenocarcinoma (A549), cisplatin-resistant A549 (A549R), human hepatoma (HepG2) and human metastatic breast cancer (MDA-MB-231) as well as human normal liver (LO2) cells by the MTT (3-(4,5-dimethylthiazol-2-yl)-2,5-diphenyltetrazoliumbromide) assay (Table 1). Both **Ir1** and **Ir2** exhibit higher anticancer activity than cisplatin. In addition, **Ir1** and **Ir2** are effective on cisplatin-resistant A549R cells, which indicates that they can overcome cisplatin resistance. **Ir1** and **Ir2** have similar lipophilicity with log *P*_o/w_ values being 1.42 and 1.58, respectively. However, **Ir1** exhibits higher cytotoxicity than **Ir2** in the cancer cells tested.

**Table 1:** IC_50_ (μM) values of **Ir1** and **Ir2** towards different cell lines^*a*^

Cell	IC_50_ (μM)		
	**Ir1**	**Ir2**	Cisplatin
HeLa	3.0 ± 0.2	6.9 ± 0.2	16.0 ± 1.2
A549	3.6 ± 0.4	5.9 ± 0.5	21.1 ± 1.5
A549R	8.5 ± 0.3	10.6 ± 0.8	124.0 ± 9.9
HepG2	6.7 ± 1.1	10.2 ± 0.8	9.1 ± 0.5
MDA-MB-231	2.1 ± 0.5	7.3 ± 0.8	16.4 ± 0.9
LO2	5.9 ± 0.4	13.3 ± 1.4	22.8 ± 2.1

^*a*^IC_50_ values are drug concentrations necessary for 50% inhibition of cell viability. Data are presented as means ± standard deviations obtained in at least three independent experiments. Cells were treated with the compounds for 48 h.

### Intracellular localization

**Fig. 4:**
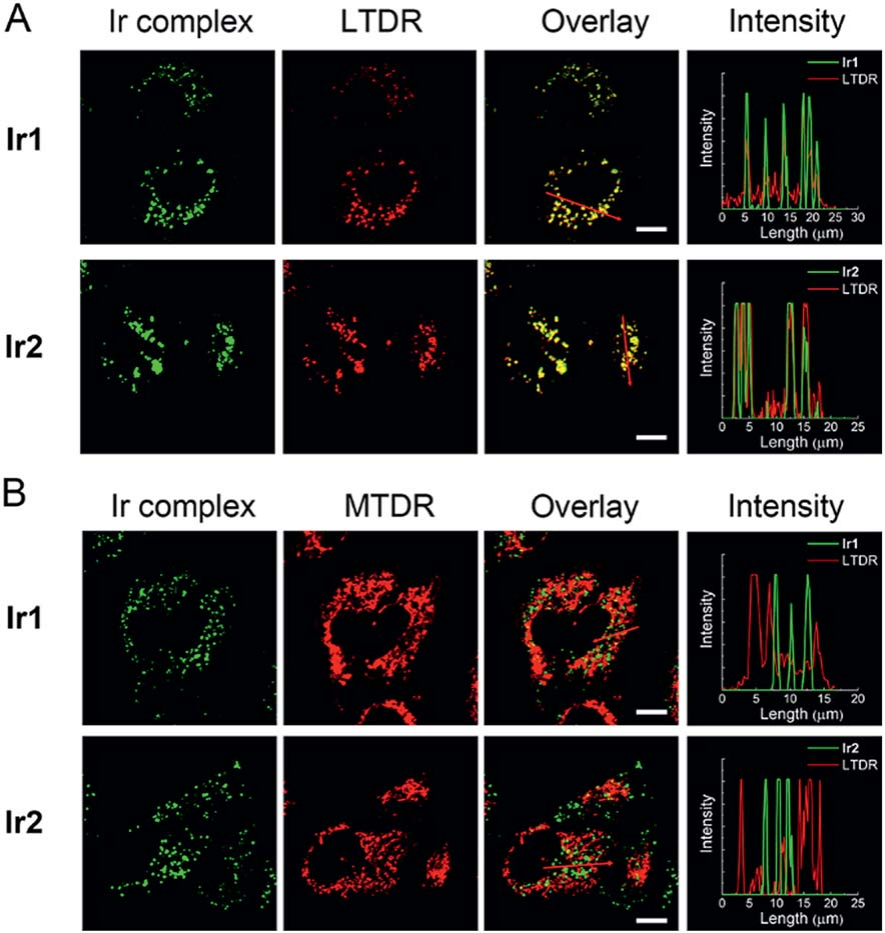
Confocal microscopy images of A549 cells costained with **Ir1** (4 μM, 2 h)/**Ir2** (4 μM, 2 h) and LTDR ((A) 50 nM, 0.5 h)/MTDR ((B) 100 nM, 0.5 h). The intensity profiles of the Ir(iii) complexes and the dyes are plotted along arrows across the A549 cells in the overlay images. Scale bars: 10 μm.

The cellular uptake levels and intracellular localization of **Ir1** and **Ir2** can be monitored by tracking the luminescence of the complexes using confocal microscopy. The complexes can enter A549 cells and emit intense dot-like green luminescence in the cytoplasm after 2 h incubation (Fig. S28†). In order to study more precisely about the subcellular localization of the complexes, we stained mitochondria and lysosomes with commercial dyes respectively before adding the complexes. The confocal images were quantified by an intensity profile (Fig. 4). The bright green spots of the complexes are clearly overlapping with the lysosomal probe (LysoTracker Deep Red, LTDR). Both complexes show high Pearson's colocalization coefficient (PCC) with LTDR (PCC**_Ir1_**_–LTDR_ = 0.81, PCC**_Ir2_**_–LTDR_ = 0.82). In contrast, barely no overlap of luminescent regions between **Ir1**/**Ir2** and the mitochondrial commercial probe (MitoTracker Deep Red, MTDR) can be observed. The PCC values of MTDR with **Ir1** and **Ir2** are 0.12 and 0.07, respectively. These results indicate that **Ir1** and **Ir2** can specifically image lysosomes.

In order to further explore the mechanism of **Ir1** and **Ir2** targeting lysosomes, we alkalized lysosomes with bafilomycin (BAF) before adding LTDR or Ir(iii) complexes (Fig. S29†). When lysosomes are alkalized, LTDR and the complexes diffusely distribute in the cells and lose their typical intracellular dot-like distribution. Therefore, the acidic environment within lysosomes is one of the necessary conditions for **Ir1** and **Ir2** to accumulate in lysosomes. The reason for **Ir1** and **Ir2** to accumulate in lysosomes may be similar to that described for LTDR. LTDR consists of a fluorophore linked to a weak base, and the protonation of weak bases can retain it within the lysosomal membrane.^48^ The pH-dependent protonation/deprotonation properties of **Ir1** and **Ir2** and their deprotonation in the cytoplasm to the basic forms may contribute to their ability to target and image acidic lysosomes.

We further used ICP-MS to quantitatively determine the intracellular localization of the complexes (Fig. S30†). The results show that most of the **Ir1** and **Ir2** remain in the cytoplasm. In addition, a small portion of **Ir1** and **Ir2** is detected in the mitochondria. This may be due to the lipophilicity of **Ir1** and **Ir2** after deprotonation, so they can be captured by mitochondria that are also lipophilic. The presence of **Ir1** and **Ir2** in mitochondria is observed by confocal microscopy, which may be ascribed to the weak fluorescence of the deprotonated forms.

### Induction of cell death

Based on the morphological characteristics and biochemical markers, cell death can be divided into several categories, mainly including necrosis, apoptosis and autophagy.^49^ First, annexin V/propidium iodide (PI) double staining was carried out to verify whether **Ir1** and **Ir2** induce apoptosis under different treatment conditions (Fig. S31†). The method can identify cells in different stages, such as viable, apoptotic, or necrotic cells.^50^ A549 cells treated with cisplatin show typical characteristics of early apoptosis (annexin V-positive and PI-negative). No obvious apoptosis features are observed in A549 cells treated with **Ir1** or **Ir2** within 24 h. However, cells treated with **Ir1** (12 μM) show significant apoptotic characteristics after 48 h treatment.

During apoptosis, the volume of cells shrinks. We further monitored the volume change of Ir(iii)-treated cells (Fig. S32†). The population of cells treated with **Ir1**/**Ir2** under relatively milder conditions is kept at a high forward scatter (FSC) and low side scatter (SSC) state. However, after incubation with **Ir1** at 12 μM for 48 h, a separate subpopulation with a low FSC and high SSC profile is formed, which shows the occurrence of apoptosis.

DNA ladder experiments and PI staining of fixed cells were performed to analyse the DNA fragmentation in nuclei. After incubation with **Ir1** at 12 μM for 48 h, about 43% of the cells display fragmented nuclei (Fig. S33†). An increase in DNA fragmentation is observed in cells treated with **Ir1** at 12 μM for 48 h, which is similar to that found in cisplatin-treated cells (Fig. S34†). However, the integrity of DNA is kept with shorter incubation periods or lower drug concentrations.

Iridium complexes can often induce cell death by producing intracellular ROS.^26^,^27^ The impact of **Ir1** and **Ir2** on ROS levels is measured by 2′,7′-dichlorodihydrofluorescein diacetate (H_2_DCF-DA) staining and flow cytometry. The nonfluorescent H_2_DCF-DA can be converted to the highly fluorescent 2′,7′-dichlorofluorescein (DCF) by cellular ROS.^51^ A concentration-dependent increase in ROS levels is observed in Ir(iii)-treated cells (Fig. S35†). The DCF fluorescence increases by about 11- and 9-fold in **Ir1**- and **Ir2**-treated (4 μM, 12 h) A549 cells, respectively. Co-treatment of Ir(iii) with the ROS scavenger (NAC) leads to marked inhibition of cell death induced by **Ir1** and **Ir2** (Fig. S36†). These results show that cell death induced by **Ir1** and **Ir2** is ROS-mediated.

Finally, we used different inhibitors to further study the modes of cell death induced by **Ir1** and **Ir2**. Treatment of **Ir1** and **Ir2** shows no impact on caspase activation, as evaluated by a Caspase-Glo® 3/7 Assay (Fig. S37†). Accordingly, the pan-caspase inhibitor z-VAD-fmk cannot diminish the cell death-inducing effects of **Ir1** and **Ir2** (Fig. S38†). Cells pre-treated with 3-methyladenine (3-MA, an autophagy inhibitor) show increased cell viability. Necrostatin-1 (Nec-1, a RIP1-specific inhibitor that can prevent necroptosis) has minimal impact on cell viability. Similarly, cycloheximide (a protein synthesis inhibitor that prevents paraptosis) does not alter the cell viability notably.^52^,^53^ These data collectively indicate that **Ir1** and **Ir2** can mainly induce ROS-mediated and caspase-independent apoptotic cell death.

### Inhibition of autophagy

**Fig. 5:**
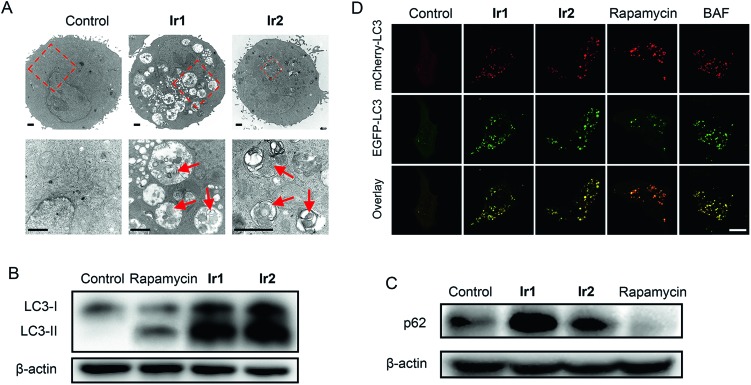
(A) Representative TEM images showing the ultra-structures of A549 cells treated with **Ir1** (4 μM) and **Ir2** (4 μM) for 24 h. Scale bars: 2 μm. (B and C) Protein expression levels of LC3 and p62 in A549 cells after treatment with **Ir1** (4 μM), **Ir2** (4 μM) or rapamycin (1 μM) for 24 h. (D) A549 cells transfected with mCherry-EGFP-LC3. The cells were examined by confocal microscopy using filters for EGFP and mCherry fluorescence. Cells were treated with **Ir1** (4 μM), **Ir2** (4 μM), rapamycin (1 μM) or BAF (200 nM) for 12 h. Scale bar: 10 μm.

For small molecule-based iridium complexes, the anti-cancer properties are influenced by many factors, *e.g.*, molecular charge, size, substituent group, hydrophobicity/hydrophilicity and subcellular localizations.^16^,^18^,^54^ Small structural modifications may lead to changes in biological activity and the mechanism of action. We used transmission electron microscopy (TEM) to observe the ultrastructural changes in cells treated with **Ir1** or **Ir2** (Fig. 5A). Compared with the control group, cells treated with **Ir1** or **Ir2** display many vacuoles containing plenty of undegraded organelles or cytoplasmic substances, whereas the nuclei remain intact. These are typical morphological features of autophagy.^55^ Besides, the vacuoles in cells treated with **Ir2** are obviously fewer than those observed in **Ir1**-treated cells.

Microtubule-associated protein (MAP) light chain 3 (LC3) and p62 protein (also called sequestosome 1, SQSTM1) can be used as markers for events related to autophagy.^56^,^57^ During autophagy, the soluble form of LC3 (LC3-I) is converted into the membrane-bound form LC3-II with a spotted distribution in the cytoplasm. When autophagy is induced and lysosomal functions are intact, an increase in LC3-II levels will be accompanied by a decrease in p62 levels. However, when autophagy is blocked due to impaired lysosomal functions, LC3-II and p62 accumulation can be observed. After A549 cells are treated with **Ir1** or **Ir2** for 24 h, a significant increase in both LC3-II and p62 levels is detected (Fig. 5B and C). However, an increase in LC3-II levels and a decrease in p62 levels are observed for rapamycin that can induce autophagy without significant effects on the lysosomes.^58^

The tandem fluorescent-tagged protein mCherry-EGFP-LC3 can inhibit autophagic flux based on different pH stability of the fluorescent proteins. EGFP is easily hydrolysed by acidic lysosomes, while mCherry is stable in acidic environments. The fluorescence intensities of mCherry and EGFP in A549 cells treated with **Ir1** and **Ir2** are similar (Fig. 5D). Similar phenomena are observed in cells treated with the alkalinizing agent BAF. In contrast, rapamycin-treated cells predominantly exhibit strong red fluorescence of mCherry. The results suggest that **Ir1** and **Ir2** can inhibit the autophagic flux.

### Alkalinization of lysosomes

Lysosomes can decompose waste delivered by autophagosomes through internal hydrolases, so lysosomal dysfunction is the main reason for the inhibition of autophagic flux.^1^ As **Ir1** and **Ir2** could inhibit the autophagic flux, we next studied their ability to affect the normal function of lysosomes. It has been reported that the chloride concentration inside lysosomes is higher than that outside lysosomes,^59^ as **Ir1** and **Ir2** are concentrated in lysosomes and have Cl^–^/HCO_3_^–^ transport ability through an anion exchange mechanism. Therefore, the complexes may transport chloride out of lysosomes along the concentration gradient and transport the HCO_3_^–^ into the lysosomes to balance the charge. As a result, **Ir1** and **Ir2** may increase the lysosomal pH by increasing the HCO_3_^–^ concentration in the lysosomes.^13^

**Fig. 6:**
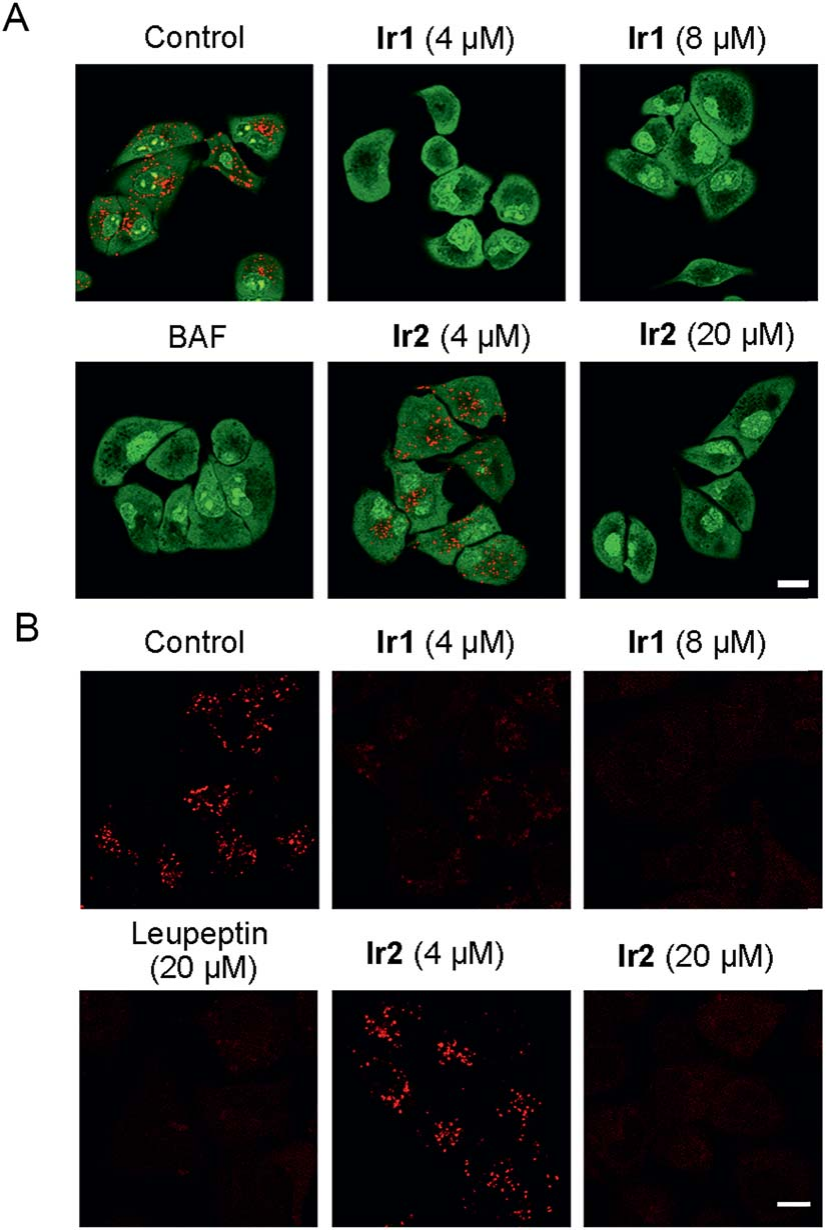
(A) A549 cells were incubated with different concentrations of **Ir1** and **Ir2** or BAF (200 nM) for 6 h at 37 °C, followed by addition of 5 μM AO for a further 1 h incubation. (B) A549 cells pretreated with **Ir1** (4 μM and 8 μM), **Ir2** (4 μM and 20 μM) or leupeptin (20 μM) for 6 h were incubated with MR-(RR)_2_ for 4 h. Scale bars: 10 μm.

First, the acridine orange (AO) staining experiment was performed to determine whether **Ir1** and **Ir2** could cause lysosomal alkalinization. AO can be protonated, concentrated and dimerized in acidic compartments, and it emits green fluorescence in the cytoplasm/nuclei and red fluorescence in lysosomes.^60^ It can be seen that the red fluorescence of AO is greatly diminished in cells treated with **Ir1** (4 μM, 6 h; Fig. 6A), which indicates that **Ir1** can effectively alkalize lysosomes. The capability of **Ir2** to alkalize lysosomes is much lower, and a much higher dose (20 μM) is needed to obtain similar effects. Accordingly, LTDR was diffusely distributed in cells and lost its ability to image lysosomes in cells pretreated with **Ir1**/**Ir2** (Fig. S39†). Next, we used a ratiometric lysosomal pH probe, fluorescein–tetramethylrhodamine-labeled dextran, to quantitatively measure the impact of **Ir1** and **Ir2** on the lysosomal pH. After treatment with **Ir1** and **Ir2**, the lysosomal pH increases from about 4.6 to 6.8 and 5.0 for **Ir1** and **Ir2**, respectively (Fig. S40–42†). These results collectively show that Ir(iii) treatment can cause lysosome alkalinisation.

It has been reported that the activity of the proteolytic enzyme cathepsin B is closely related to lysosomal pH.^61^ Then we measured the cathepsin B activity by using a Magic Red cathepsin detection kit. The cell permeable probe is non-fluorescent, and it emits red fluorescence when the peptide linkage of the probe is cleaved by cathepsin B in lysosomes. A concentration-dependent decrease in the MR-(RR)_2_ fluorescence is observed in Ir(iii)-treated cells (Fig. 6B). The red fluorescence is significantly diminished in cells treated with relatively higher doses of **Ir1** (8 μM) and **Ir2** (20 μM), which indicates the loss of cathepsin B activity. A similar phenomenon is also observed in cells treated with leupeptin (a serine and cysteine protease inhibitor). As **Ir1** and **Ir2** can alkalize lysosomes and inactivate hydrolases, they can cause lysosomal dysfunction and block autophagy at the lysosomal stage.

### *In vivo* anticancer activity

**Fig. 7:**
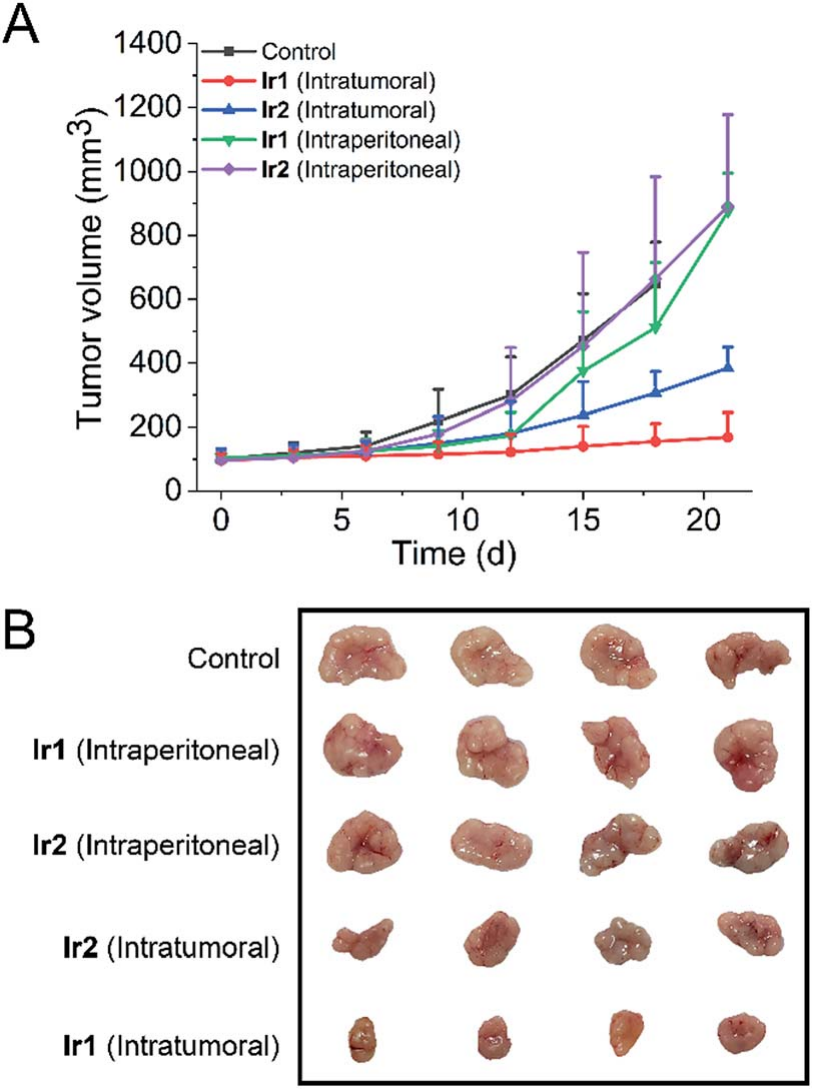
Tumor volumes (A) and tumor images (B) of each group after 3 weeks (*n* = 4). **Ir1** and **Ir2** were dissolved in PET (6% poly(ethylene glycol) 400, 3% ethanol, 1% Tween 80, and 90% PBS), and mice were treated with Ir(iii) complexes by intratumoral injection or intraperitoneal injection every 3 d (50 μL, 10 mg kg^–1^).

To test whether the iridium complexes can inhibit tumor growth *in vivo*, we used nude mice implanted with A549 cells (Fig. 7). Mice were divided into five groups (control group, two intratumoral injection groups and two intraperitoneal injection groups). For the intratumoral injection groups, both complexes can inhibit tumor growth and **Ir1** exhibits a significantly higher tumor inhibitory effect than **Ir2**. After three weeks, the tumor volume of **Ir1**-treated mice decreases by about 75% compared with the control group. No significant change in the body weight of mice is observed in both **Ir1**- and **Ir2**-treated mice (Fig. S43†).

In addition, H&E staining was performed on excised major organs, including the heart, lung, liver, spleen, and kidney, after intratumoral administration of the complexes. Similarly, no significant abnormalities are observed in the images of the stained organ slices (Fig. S44†). These results suggest that **Ir1** and **Ir2** have low systemic toxicity.

However, compared with the control group and intratumoral injection groups, no significant therapeutic effect was observed for the intraperitoneal injection groups. This may be attributed to the fact that the complexes do not have obvious tumor-targeting ability. These results indicate that **Ir1** and **Ir2** process potent *in vivo* anticancer activities and low systemic toxicity, but structural optimization is still needed to achieve improved tumor-targeting capacities.

## Conclusions

**Fig. 8:**
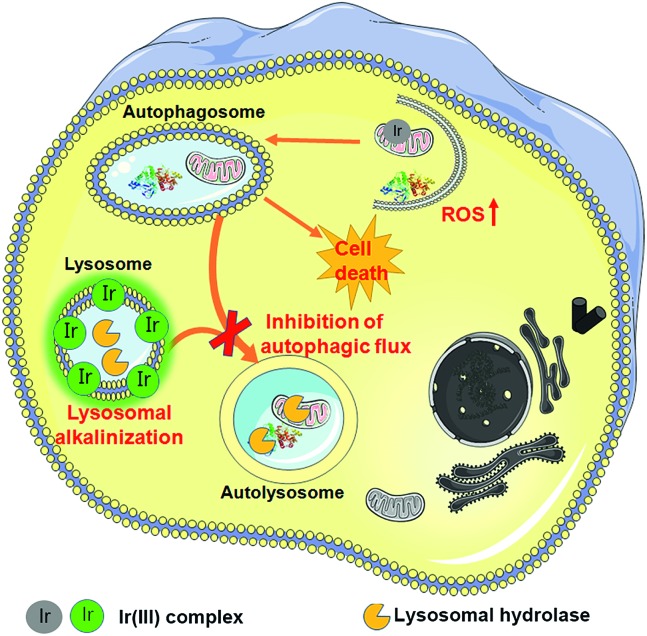
Schematic illustration of the mechanism of action of **Ir1** and **Ir2**. First, **Ir1** and **Ir2** induce autophagy by causing an increase in intracellular ROS. Damaged mitochondria or proteins are swallowed in autophagosomes. **Ir1** and **Ir2** can accumulate in lysosomes and specifically image lysosomes. Besides, **Ir1** and **Ir2** can alkalinize lysosomes through anion disturbance and inhibit the fusion between autophagosomes and lysosomes.

In summary, we have synthesized two cyclometalated iridium(iii) complexes, **Ir1** and **Ir2**, as anion transporters. Both **Ir1** and **Ir2** can promote anion transport in liposomal models. Both complexes exhibit higher *in vitro* anticancer activities than cisplatin against the cancer cells screened. **Ir1** and **Ir2** can accumulate in lysosomes and specifically image lysosomes. **Ir1** and **Ir2** mainly induce cell death through apoptosis by elevating intracellular ROS. Interestingly, **Ir1** and **Ir2** can increase lysosomal pH and impair the activity of lysosomal enzymes possibly through promoting chloride transport (Fig. 8). **Ir1** displays higher potency than **Ir2** in lysosomal alkalinization, *in vitro* cytotoxicity, autophagy induction and autophagic flux inhibition. Moreover, **Ir1** displays a good anticancer effect and undetectable systemic toxicity *in vivo*. Our future study will be concentrated on the structural optimization of metal complexes to achieve better tumor-targeting performance. In all, this study provides new insights into the mechanism investigations of metallo-anticancer drugs and may aid in the future rational design of new types of anion transporters.

## Conflicts of interest

There are no conflicts to declare.

## Supplementary Material

Supplementary information

Crystal structure data
