# Supplementary material for: Inhibition of autophagic flux by cyclometalated iridium(iii) complexes through anion transportation
Source: Chem Sci. 2019 Jan 31;10(11):3315–23. doi: 10.1039/c8sc04520h (PMC6428141; doi:10.1039/c8sc04520h)
Supplement: Supplementary file 1 [file SC-010-C8SC04520H-s001.pdf]

Electronic Supplementary Material (ESI) for Chemical Science.
This journal is © The Royal Society of Chemistry 2019

Inhibition of Autophagic Flux by Cyclometalated Iridium(III) Complexes through Anion Transportation

Mu-He Chen,†[a] Yue Zheng,†[a] Xiong-Jie Cai,†[b] Hang Zhang,[a] Fang-Xin Wang,[a] Cai-Ping Tan,*[a] Wen-Hua Chen,*[b] Liang-Nian Ji[a] and Zong-Wan Mao*[a]

Supporting Information

Table of Contents

Supporting Materials and Methods……………………………………………..S2

Supporting Figures and

Tables…………………………………………………S11

Supporting References..S36

Supporting Materials and Methods

General materials and methods

Iridium chloride hydrate (Alfa Aesar, USA), ppy (2-phenylpyridine, Sigma Aldrich, USA), NH_4PF_6, egg-yolk L-α-phosphatidylcholine (EYPC), NaCl, $NaNO_3$, DMSO, chloroform, triton X-100, 3-(4,5-dimethylthiazol-2-yl)-2,5-diphenyltetrazolium bromide (MTT), octanol, lysotracker deep red (LTDR), mitotracker deep red (MTDR), bafilomycin (BAF) and acridine orange (AO) were purchased from commercial resources and used as received. Avanti's mini-extruder (Avanti Polar Lipids, Inc., Alabaster, Alabama, USA), nuclepore track-etched polycarbonate membranes (100 nm) (Whatman, Florham Park, New Jersey, USA), chloride ion selective electrode (Mettler-Toledo PerfectIon). H_2biim (2,2′-biimidazole) and Hpyim (2-(1H-imidazol-2-yl)pyridine) was synthesized by literature procedures.[1,2] All the solvents were purified and degassed by standard procedures. **Ir1** and **Ir2** were dissolved in DMSO immediately before the cell experiments, and the final concentration of DMSO was maintained at 1% (v/v).

NMR spectra were recorded on a Bruker Avance 400 MHz spectrometer (Germany) or on a Varian Mercury Plus 300 MHz spectrometer (USA). Shifts were referenced relative to the internal solvent signals. ESI-MS spectra were obtained on a Thermo Finnigan LCQ DECA XP spectrometer (USA) in positive mode. The quoted *m/z* values indicated the major peaks in the isotopic distribution. Microanalysis (C, H, and N) was performed with an Elemental Vario EL CHNS analyzer (Germany). The UV/Vis spectra were measured in degassed solvents by a Varian Cary 300 spectrophotometer (USA). The emission measurements were performed in degassed solvents using a FLS980 combined fluorescence lifetime and steady-state spectrometer (UK). Quantum yields at room temperature were determined according to literature methods using $[Ru(bpy)_3](PF_6)_2$ as the reference standard.[3]

Synthetic protocols and characterizations

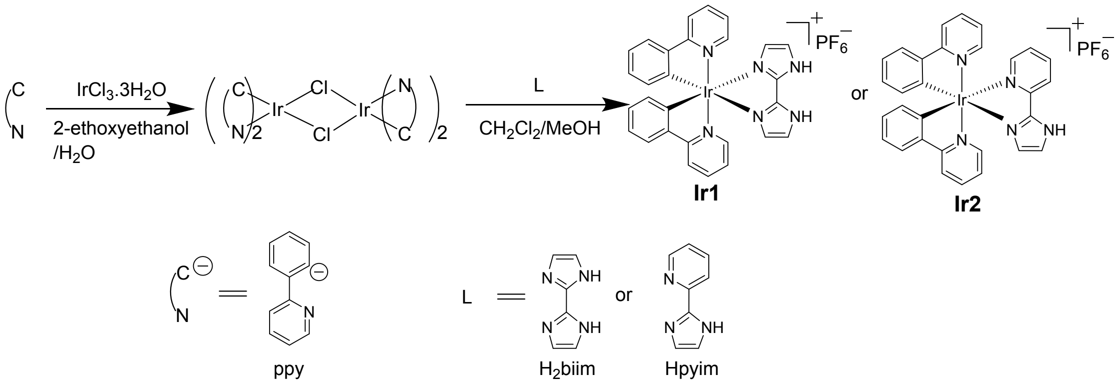

Scheme S1 Synthetic procedure of the Ir(III) complexes.

Synthetic procedure of Ir1 and Ir2: The $IrCl_3{\cdot}3H_2O$ (1 equiv) and the ppy ligand

(2.1 equiv) were mixed, then added into 2-ethoxyethanol/H_2O (3:1, v/v), After heating under reflux for 24 h, it was cooled to room temperature and the precipitate was filtered off. The precipitate was washed with water, methanol and hexane, respectively, and then dried in vacuum. The product was used directly in the next step without further purification. In a mixed solvent of CH_2Cl_2/CH_3OH (2:1, v/v), the Ir(III) μ-chloro-bridged dimer $[Ir(ppy)_2Cl]_2$ (1 equiv.) and the corresponding ligand (H_2biim or Hpyim, 2 equiv) were added. The mixture was heated under reflux in a nitrogen atmosphere. After 6 h, the mixture was cooled to room temperature and the reaction mixture was evaporated to dryness under reduced pressure. The product was redissolved with methanol and an acid aqueous solution containing 6-fold excess of NH_4PF_6 was added. The resulting precipitate was filtered off, washed by *n*-hexane and purified by column chromatography on silica gel eluted with CH_2Cl_2/ CH_3OH (50:1, v/v). The crystals were obtained by diffusion of diethyl ether into a CH_2Cl_2 solution.

[Ir(ppy)$_2$(H$_2$biim)](PF$_6$) (**Ir1**). Complex **Ir1** was obtained as a yellow powder. Yield: 0.277 g (71.1%). ^{1}H NMR (400 MHz, DMSO-d_6) δ 14.12 (s, 2H), 8.20 – 8.13 (m, 2H), 7.91 – 7.78 (m, 4H), 7.69 (dd, J = 5.9, 1.4 Hz, 2H), 7.33 (d, J = 1.4 Hz, 2H), 7.19 (ddd, J = 7.3, 5.8, 1.4 Hz, 2H), 6.91 (td, J = 7.5, 1.3 Hz, 2H), 6.80 (td, J = 7.4, 1.3 Hz, 2H), 6.34 (d, J = 1.3 Hz, 2H), 6.27 (dd, J = 7.6, 1.2 Hz, 2H). ^{13}C NMR (101 MHz, DMSO) δ 167.95, 150.46, 149.15, 144.92, 141.87, 138.23, 131.91, 129.72, 126.67, 124.82, 123.73, 122.24, 121.57, 119.66. ESI-MS (CH_2Cl_2): m/z calcd for $[M-PF_6]^+$, 634.7; found: 635.2. Elemental analysis: calcd (%) for $C_{28}H_{22}F_6IrN_6P$: C, 43.13; H, 2.84; N, 10.78; found C, 47.63; H, 3.11; N, 11.94. HPLC purity: t_R = 19.53, 97.95%.

[Ir(ppy)$_2$(Hpyim)](PF$_6$) (**Ir2**). Complex **Ir2** was obtained as a yellow powder. Yield: 0.321 g (75.4%). ^{1}H NMR (400 MHz, DMSO-d_6) δ 8.35 (d, J = 7.9 Hz, 1H), 8.26 – 8.17 (m, 3H), 7.95 – 7.84 (m, 4H), 7.77 – 7.61 (m, 4H), 7.53 (ddd, J = 7.2, 5.5, 1.2 Hz, 1H), 7.19 (dddd, J = 16.4, 7.3, 5.8, 1.4 Hz, 2H), 6.98 (dtd, J = 16.1, 7.5, 1.2 Hz, 2H), 6.86 (dtd, J = 21.9, 7.4, 1.3 Hz, 2H), 6.51 (d, J = 1.3 Hz, 1H), 6.23 (ddd, J = 23.7, 7.6, 1.1 Hz, 2H). ^{13}C NMR (101 MHz, DMSO) δ 167.63, 167.47, 152.08, 150.39, 149.46, 149.19, 148.45, 147.75, 144.74, 144.46, 140.23, 138.97, 138.74, 131.77, 131.69, 130.51, 129.99, 127.76, 127.40, 125.42, 125.01, 124.15, 123.31, 122.40, 122.21, 122.16, 120.22, 120.01. ESI-MS (CH_2Cl_2): m/z calcd for $[M-PF_6]^+$, 645.7; found: 646.2. Elemental analysis: calcd (%) for $C_{30}H_{23}F_6IrN_5P$: C, 45.57; H, 2.93; N, 8.86; found C, 45.52; H, 2.96; N, 8.92. HPLC purity: t_R = 20.62, 98.65%.

Purity analysis

The purity analysis was performed by an Agilent Technology 1260 Infinity HPLC system using a C18 column (APursuit XRs, 10 μm, 21.2 mm × 250 mm). We used water as mobile phase A and methanol as mobile phase B. The flow rate of the mobile phase was controlled at 10 mL/min and the injection volume of the sample was 30 μL. Elution and conditions were the following: at 0 min, 90% phase A + 10% phase B; at 5 min, 75% phase A + 25% phase B; at 10 min, 25% phase A + 75% phase B; at 20 min, 10% phase A + 90% phase B. Peaks were detected at 280 and 360 nm.

Stability in human plasma

The plasma stability experiment was performed by a reported procedure.[4] 800 μM of diazepam stock solution (internal standard, obtained from Sigma-Aldrich) was first prepared. A 12.5 μL portion of the solution containing the iridium complexes (20 μM) to be studied and 12.5 μL of the diazepam solution were added to 975 μL of plasma. The resulting human plasma solution was incubated for 72 h at 37 °C with continuous and gentle shaking (~300 rpm). Afterward, 3 mL of methanol/CH_2Cl_2 mixture was added to the plasma solution, and the mixture was shaken for 20 min at room temperature and finally centrifuged at 2000 × g at 4 °C for 30 min. Finally, the organic layer was separated from the water phase, and the solvent was evaporated. The resulting residue was suspended in 200 μL of 1:1 acetonitrile/H_2O (v/v). The suspension was filtered and analyzed by LC–UV. A total of 0.1 μL of the solution was injected into an HPLC instrument (Thermo, USA) connected to a mass spectrometer (TSQ Quantum Ultra, Thermo, USA) operating in ESI mode. A Hypersil Gold Dim (100 mm × 2.1 mm, Thermo, USA) reverse phase column was used with a flow rate of 0.4 mL/min. The runs were performed with a linear gradient of A (acetonitrile, Sigma-Aldrich HPLC grade) in B (distilled water containing 0.1% HCOOH).

Crystallographic structure determination

X-ray diffraction measurements were performed on a Bruker Smart 1000 CCD diffractometer with Mo and Cu Kα radiation (λ= 0.71073 or 1.54184 Å) at 120(2) and 292(1) K for **Ir1** and **Ir2**, respectively. The structures of **Ir1** and **Ir2** were solved by direct methods using the program SHELXS and refined by the full-matrix least-squares on F^2 with the program SHELXL.[5] Details of crystallographic data, data collection and structure refinements are summarized in Table S1. Selected bond distances and angles are listed in Table S2. Thermal ellipsoids in structural plots of **Ir1** and **Ir2** are plotted at a 50% probability level using Diamond 3.2.

Protonation/deprotonation processes of complexes

The DMSO solutions of **Ir1** and **Ir2** were diluted with buffer solutions at different pH values to a final concentration of 20 μM. After **Ir1** and **Ir2** was excited at 405 nm, fluorescence spectra were collected with FLS980. The pK_a values of **Ir1** and **Ir2** were calculated from a plot of emission intensity *vs* pH as described in previous study.[6]

Anion transport assays

Vesicles used for anion transport assays were prepared according to a previous protocol.[7] EYPC (20 mg) was dissolved in chloroform (0.5 mL) in a pyrex test tube and the chloroform was then evaporated under a stream of nitrogen to give a thin film of lipid. The lipid film was further dried at room temperature and vacuum for at least 4 h. After adding 500 mM NaCl and 5mM citric-phosphate buffer in different pH (4.0, 5.0,

6.0 or 7.2) to the lipid membrane, vortex vigorously for 1 min. The mixture was incubated for 5 min at room temperature and then continued to vortex for 1 min before another 20 min incubation. The suspension was then subjected to eight freeze/thaw cycles (77 K/325 K), followed by extrusion (15 times) through a 100 nm Nuclepore membrane using an Avanti's Mini-Extruder (Avanti Polar Lipids, Inc., Alabaster, Alabama, USA). After extrusion, the mixture was incubated for 1 h at room temperature. The unencapsulated chloride was removed *via* gel filtration (Sephadex G-25, eluted with corresponding 500 mM $NaNO_3$ and citric-phosphate buffer in different pH (4.0, 5.0, 6.0 or 7.2)). Finally, vesicles are dispersed with the solution used to elute them to reach a known volume. A chloride ion selective electrode is used to monitor the chloride efflux in the solution. After 5 min, 50 μL of 5 wt% aqueous Triton X-100 was added. The relative chloride efflux was calculated using the following equation:

$$\text{Relative chloride efflux (\%)} = \frac{[Cl^-] - [Cl^-]_0}{[Cl^-]_{total} - [Cl^-]_0} \times 100$$

$[Cl^-]_0$ represent the concentrations of chloride ion at the initial time.

$[Cl^-]$ represent the concentrations of chloride ion after a period of time.

$[Cl^-]_{total}$ represent the concentrations of chloride ion after the addition of 5 wt% aqueous Triton X-100.

To compare the initial rate of chloride release (k_{ini}) of different complexes at the same concentration, we performed a non-linear fitting analysis for **Ir1** with the asymptotic function $y=a-b\cdot c^x$ using Origin 2018, where y is the relative chloride efflux (%) and x is time (s). k_{ini} is calculated from $k_{ini} = -b\cdot ln(c)$ (obtained in $\%\cdot s^{-1}$). For **Ir2**, k_{ini} is calculated by fitting the obtained chloride efflux to $y = a+b\cdot x$, where y is the relative chloride efflux (%) and x is time (s). k_{ini} is calculated by the slope b.

Hill plots were constructed as described above for various concentrations of carrier. The chloride efflux (%) 260 s after the addition of carrier is plotted as a function of the carrier concentration. Data points can then be fitted to the Hill equation using Origin 2018:

$$y = V_{min} + (V_{max} - V_{min})\frac{x^n}{k^n + x^n}$$

where y is the relative chloride efflux at 260 s (%) and x is the carrier concentration (μM). V_{max}, V_{min}, k and n are the parameters to be fitted. V_{max} is the maximum efflux possible, V_{min} is the minimum efflux possible, n is the Hill coefficient and k is the carrier concentration needed to reach $V_{max}/2$. From the Hill plot it is therefore possible to obtain $EC_{50,260s}$ values, defined as the carrier concentration (μM) needed to obtain 50% chloride efflux after 260s.

Measurement of intracellular Cl^- concentrations

For measurements of the time-dependent influx of Cl^- into cells, A549 cells were incubated with culture media added with 10 mM MQAE for 1 h at 37 °C. After washing with PBS, A549 cells were treated with 10 μM of **Ir1** or **Ir2** in culture media

for 1, 2 and 3 hours at 37 °C. The MQAE fluorescence was measured using a microplate reader (λ_{ex} = 350 nm, λ_{em} = 460 nm).

Lipophilicity

The lipophilicity of the iridium(III) complexes, which was presented as log$P_{o/w}$ values, was determined according to a reported procedure.[8] Log$P_{o/w}$ is defined as the logarithmic ratio of Ir(III) concentration in n-octanol to that in the aqueous phase.

Cell lines and culture conditions

HeLa, A549, A549R, HepG2, MDA-MB-231 and LO2 cells were purchased from Experimental Animal Centre of Sun Yat-Sen University (Guangzhou, China). The Cells were grown in DMEM (Dulbecco's modified Eagle's medium, Gibco BRL) or RPMI 1640 (Roswell Park Memorial Institute 1640, Gibco BRL) medium with 10% FBS (fetal bovine serum, Gibco BRL), 100 μg/mL streptomycin, and 100 U/mL penicillin (Gibco BRL) in a humidified incubator at 37 °C and 5% CO_2. A549R cells were exposed to increasing concentrations of cisplatin to maintain the resistance. In each experiment, cells treated with DMSO (1%, v/v) were used as vehicle-treated control.

Cell viability assays

The cytotoxicity of the tested compounds towards the indicated cell lines was determined by the MTT assay. Briefly, the cells were seeded in 96-well tissue culture plates. After overnight incubation, the cells were treated with varies concentrations of the compounds. The tested compounds were dissolved in DMSO (1%, v/v) and further diluted with fresh media immediately. After co-incubation for 44 h, 20 μL MTT (5 mg/mL) was added to each well and incubated for another 4 h. After that, the medium was removed and the formazan product was dissolved in 150 μL DMSO per well. The cell viability was evaluated by absorbance at 595 nm (Infinite F200, Tecan, Switzerland). IC_{50} values were calculated from curves constructed by plotting cell survival (%) versus compound concentration (μM).

Intracellular localization of the complexes

After incubation of A549 cells in confocal dishes for 24 h, **Ir1** and **Ir2** (4 μM) was added and incubated for an additional 2 h. Confocal microscopy (LSM 710, Carl Zeiss) was then used to observe the intracellular distribution of the Ir complexes (λ_{ex} = 405 nm, λ_{em} = 500 ± 25 nm). In order to further analyze the cellular localization of **Ir1** and **Ir2**, the cells were pretreated with the commercial lysosomal probe LTDR (50 nM) or mitochondrial probe MTDR (100 nM) (LTDR and MTDR: λ_{ex} = 633 nm, λ_{em} = 665 ± 20 nm) for 30 min, washed the cells three times with culture media and then incubated with **Ir1** and **Ir2** (4 μM) for another 2 h.

The BAF assay

A549 cells were cultured in confocal dishes for 24 h and then treated with bafilomycin (BAF, 200 nm) for 1 h before addition of the Ir complexes (4 μM, 2 h) or LTDR (50 nM, 0.5 h). A confocal microscope (LSM 710, Carl Zeiss) was then used to observe the intracellular distribution of the Ir complexes or LTDR.

ICP-MS measurement

In order to quantitatively measure intracellular localization of the complexes, we measured intracellular iridium content by using a slightly modified method reported by our lab.[9] Briefly, A549 cells were incubated in 100 mm tissue culture dishes for 24 h. After 24 h, the medium containing **Ir1** (10 μM) or **Ir2** (10 μM) was replaced. After 2 h, the cells were collected in PBS (3 mL) and were equally divided into two and counted separately.

According to the manufacturer's instructions, we used the mitochondrial isolation kit (89874, Thermo Scientific) to isolate mitochondria from Ir (III) -treated cells. The cells were centrifuged and the cell pellet was resuspended in reagent A (800 μL) and then incubated on ice for 2 min. After addition of reagent B (10 μL), incubation was continued on ice for 5 min. After reagent C (800 μL) was added, the suspension was centrifuged at 4 °C for 10 min at 700 *g*. The resulting supernatant was transferred to another tube and centrifuged at 12000 *g* for 15 min at 4 °C to obtain mitochondrial precipitates and cytosolic supernatants. The mitochondrial pellet was resuspended in reagent C (500μL) and the resulting suspension was centrifuged at 12000 *g* for 5 min to obtain purified mitochondria.

According to the instructions, we used nuclear protein extraction kit (C500009, Sangon Biotech, China) to extract nuclear components. The cells were centrifuged and resuspended in Hypotonic Buffer (600μL) and incubated on ice for 10 min. Vortex for 10 s. The cell suspension was centrifuged at 800 *g* for 5 min at 4 °C, and the resulting pellet was resuspended in Hypotonic Buffer (400 μL). Then, the suspension was centrifuged at 2500 g for 5 min at 4 °C to precipitate the nuclei components.

These different cell fractions were lysed with radio immunoprecipitation assay (RIPA) buffer. To the lysates of the different cellular components (mitochondria, cytoplasm and nuclear fraction), HNO_3 (65%, 1 mL) was added. Then incubated at room temperature for 24 h to digest completely. The mixture was diluted to a final volume of 10 mL with Milli-Q water (containing 10 ppb indium as an internal standard). The concentration of iridium was measured using an XSERIES 2 ICP-MS.

Transmission electron microscopy

A549 cells were treated with complexes for 24 h, the cells were collected, added with 2.5% glutaraldehyde in phosphate buffer (pH 7.4), and fixed overnight at 4 °C. The next day, cells were treated with osmium tetroxide, stained with uranyl acetate and lead citrate and observed under transmission electron microscopy (JEM 100 CX, JEOL, Tokyo, Japan). Use the Eversmart Jazz program (Scitex) to capture images.

The eGFP-LC3 analysis

The eGFP-LC3 vectors was transfected into A549 cells using Lipofectamine 3000 (Life Technologies, USA). Cells were transfected with eGFP-LC3 and then treated with rapamycin (1 μM), **Ir1** (4 μM) or **Ir2** (4 μM) for 12 h. Cells were fixed with 4% paraformaldehyde for 30 min and then washed twice with cold PBS. The cells were photographed with confocal microscopy (LSM 710, Carl Zeiss, Göttingen, Germany). Counts were performed with a minimum of 200 eGFP-LC3-positive cells. Cells with more than 5 dots are considered to be autophagy.

Western blotting

A549 cells were cultured in 60 mm tissue culture dishes (Corning) for 24 h and then treated with the indicated concentrations of the tested compound for 24 h. The cells were collected and washed twice with cold PBS. The cells were then lysed by addition of a radioimmunoprecipitation assay (RIPA) buffer supplemented with a protease inhibitor (Roche Diagnostics GmbH, Germany) and a phosphatase precursor sodium orthovanadate inhibitor (Sigma Aldrich). The protein concentration was quantitated by BCA reagent (Novagen Inc, USA). After loading the equal amount of protein on an SDS-polyacrylamide gel, the proteins were separated by electrophoresis, and the proteins on the gel were transferred to a polyvinylidene difluoride membrane (Millipore, USA). The membrane was blocked with 5% nonfat dry milk at room temperature for 2 h. Followed by incubation at 4 °C overnight. The appropriate HRP-conjugated secondary antibody was incubated with the membranes for 1 h at room temperature. The signal was detected using an enhanced chemiluminescence (ECL) kit (Amersham Inc, USA). Images were captured on FluorChem M (ProteinSimple, Santa Clara, CA) and analyzed manually using AlphaView software (ProteinSimple, CA, USA).

Measurement of intracellular ROS

A549 cells were treated with **Ir1** or **Ir2** at the indicated concentrations for 12 h. Cells were then harvested, washed twice with PBS, and replaced with serum-free RPMI 1640. The cells were then incubated with 10 μM H_2DCF-DA for 15 min in the dark. The fluorescence intensity of the cells was immediately measured by flow cytometry. The excitation wavelength was 488 nm and the emission wavelength was 530 nm. Green mean fluorescence intensities were analyzed by FlowJo VX software.

Acridine orange (AO) staining

After A549 cells were seeded in confocal dishes for 24 h, different concentrations of **Ir1** and **Ir2** were added and incubated for 6 h. Each dish was incubated with 5 μM AO for 1 h and then washed three times with PBS. AO has an excitation wavelength of 488 nm and emission wavelengths at 515–545 nm (green) and 610–640 nm (red). Confocal images were collected with a confocal microscope (LSM 710, Carl Zeiss).

Measurement of lysosomal pH

A549 cells were treated with 1 mg/mL fluorescein-tetramethylrhodamine-labeled dextran (70,000 MW, Invitrogen) for 12 h and then **Ir1** and **Ir2** were added for further incubation for 12 h. Cells were washed three times with PBS prior to taking image from a confocal fluorescence microscope (LSM 710, Carl Zeiss). Fluorescein is excited at 488 nm and tetramethylrhodamine (TMR) is excited at 543 nm. Fluorescein emission in the 500–550 nm band and TMR emission in the 590–700 nm band is collected. In order to obtain the pH titration curve of fluorescein-TMR labeled dextran, the cells were treated with fluorescein-TMR-labeled dextran for 12 h and then treated with different pH calibration buffers (120 mM KCl, 20 mM NaCl, 1 mM $CaCl_2$, 1 mM $MgCl_2$, 10 μM nigericin, 10 μM valinomycin, 10 mM HEPES with the pH adjusted from 4 to 8) for 30 min. The pH was measured by the intensity ratio of TMR (red) and fluorescein (green) in each lysosome. An Image-Pro Plus 6.0 software (Media Cybernetics) was used to calculate the area integral intensities for each fluorophore in each lysosome and calculate their red:green intensity ratio. The fluorescence intensity ratio was plotted against pH values.

Determination of cathepsin B activity

Cathepsin B activity was analyzed using Magic Red cathepsin detection kit-MR-$(RR)_2$ (Immunochemistry Tech, Bloomington, USA) according to the manufacturer's instructions. Briefly, after A549 cells were incubated in confocal dishes for 24 h, they were treated with different concentrations of **Ir1**, **Ir2** and leupeptin for 6 h, followed by the addition of 1x MR-$(RR)_2$ for 4 h. Cells were washed three times with PBS before confocal imaging. Confocal images were collected with a confocal microscope (LSM 710, Carl Zeiss). λ_{ex} = 543 nm, λ_{em} = 630 ± 20 nm.

Annexin V/PI apoptosis assay

Analysis was performed according to the manufacturer’s (Sigma Aldrich, USA) protocol. First, A549 cells were seeded into 6-well plates and then exposed to the indicated concentrations of the test compound for 24 h. Cells were then harvested and washed twice with cold PBS. Cells were then resuspended in 500 μL of annexin-binding buffer and then stained with 5 μL of annexin V and 10 μL PI for 15 min at room temperature and immediately analyzed by flow cytometry (FACSCalibur, Becton Dickinson, NJ, USA). The resulting histograms were analyzed using FlowJo VX software (Tree Star, OR, USA). Annexin V-FITC: λ_{ex} = 488 nm, λ_{em} = 520 ± 20 nm; PI: λ_{ex} = 488 nm, λ_{em} = 620 ± 20 nm.

Nuclear fragmentation measured by PI staining

Nuclear fragmentation was tested by flow cytometry after PI staining. Briefly, A549 cells were exposed to the indicated concentrations of the test compound for 24 h. After incubation, the cells were collected and fixed with 70% ethanol. After stored at −20 ºC overnight, the cells were centrifuged and washed twice with ice-cold PBS. The cells were then resuspended in 200 μL staining solution containing PI (10 μg/mL) and DNase-free RNase (100 μg/mL), and analyzed by flow cytometry (FACSCalibur,

Becton Dickinson, NJ, USA). Data were analyzed with ModFit LT (5.0) software (Variety Software House, Inc., ME, USA). λ_{ex} = 488 nm, λ_{em} = 620 ± 20 nm.

DNA ladder

A549 cells were incubated with **Ir1**, **Ir2** or cisplatin at the indicated concentrations for different time intervals at 37 °C. The genome DNA was extracted and purified by a commercial spin column quantification kit (AxyPrep Blood gDNA MiniPrep kit, Corning, USA) according to the protocol. Samples were loaded on a 1.5% agarose gel and were subjected to electrophoresis at 100 V for 30 min in TBE (Tris/Borate/EDTA) buffer (0.5X). The DNA ladder was stained with 2 μL Gel-Red and the images were captured on FluorChem M (ProteinSimple, Santa Clara, CA).

Caspase-3/7 activity assay

Caspase-3/7 activity was measured using the Caspase-Glo® Assay kit (Promega, Madison, WI, USA) according to the manufacturer's instructions. Cells cultured in 96 well plates were treated with **Ir1**, **Ir2** or cisplatin for different time at the indicated concentrations. 100 μL of Caspase Glo® 3/7 reagent was added to each well containing 100 μL culture medium. The mixture was incubated at room temperature for 1 h and then luminescence was measured using a micro-plate reader (Infinite M200 Pro, Tecan, Switzerland).

Evaluation of antitumor activities *in vivo*

Statement: All animal procedures were performed in accordance with the Guidelines for Care and Use of Laboratory Animals of Sun Yat-Sen University and the experimental protocols were approved by the Sun Yat-Sen University Animal Care and Use Committee.

Female BALB/c-(nu/nu) nude mice aged 4–5 weeks were purchased and bred in the Center of Experiment Animals at the Sun Yat-Sen University. A549 cells (4×10^6) were suspended in 100 μL PBS, and the xenografts were established by subcutaneous injection. When the tumor grew to about 100 mm^3, the nude mice were randomly allocated into 7 groups (n = 4) before the experiment. As for the vehicle control group, each mouse was injected with 100 μL PET diluent (6% poly(ethylene glycol) 400, 3% ethanol, 1% Tween 80, and 90% PBS). **Ir1** and **Ir2** were also dissolved in PET, and mice were treated with Ir(III) complexes by intratumoral injection or intraperitoneal injection every 3 d (50 μL, 10 mg kg^{-1}). Besides, we used a nanoprecipitation method to encapsulate the complexes within a PLGA-b-PEG block copolymer. Two groups of Mice were treated with the encapsulated nanoparticles by intraperitoneal injection every 3 d (10 mg kg^{-1}). The tumor sizes and body weights were measured every 3 d. The tumor volume was calculated by the formula $V = ab^2 \times 0.52$, where a and b were the longest and shortest diameters of the tumor separately. After the experiment, the

mice were sacrificed and tumors were separated.

Supporting Figures and Tables

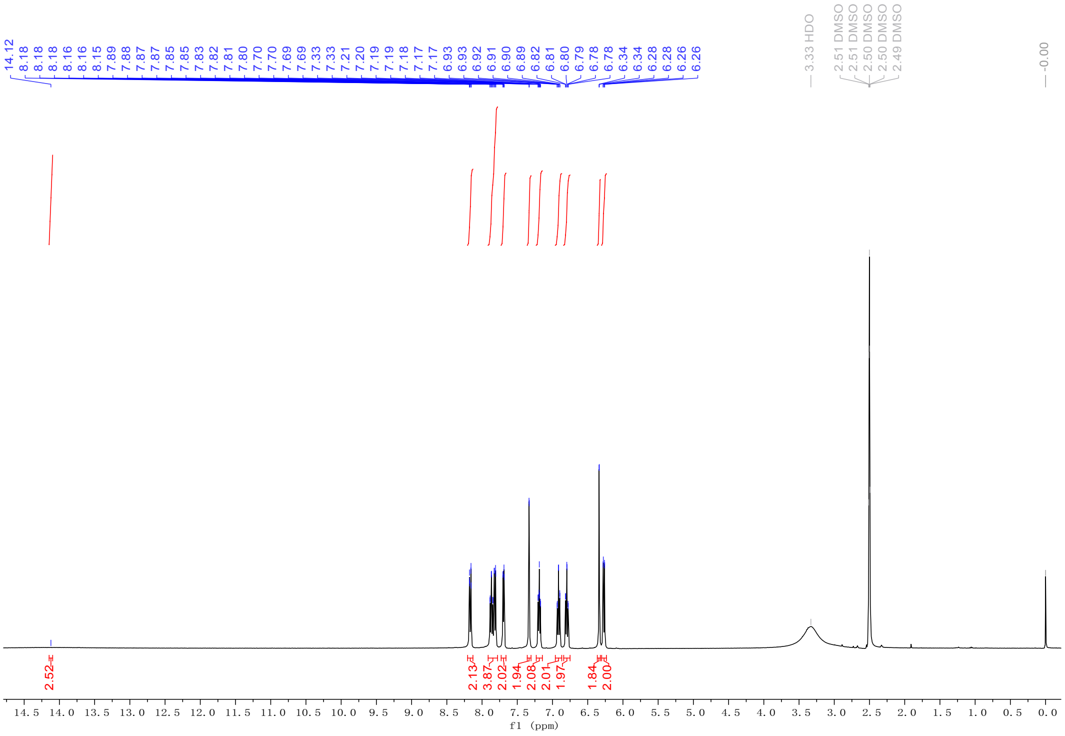

Fig. S1 ^{1}H NMR spectrum of **Ir1** in DMSO-d_6.

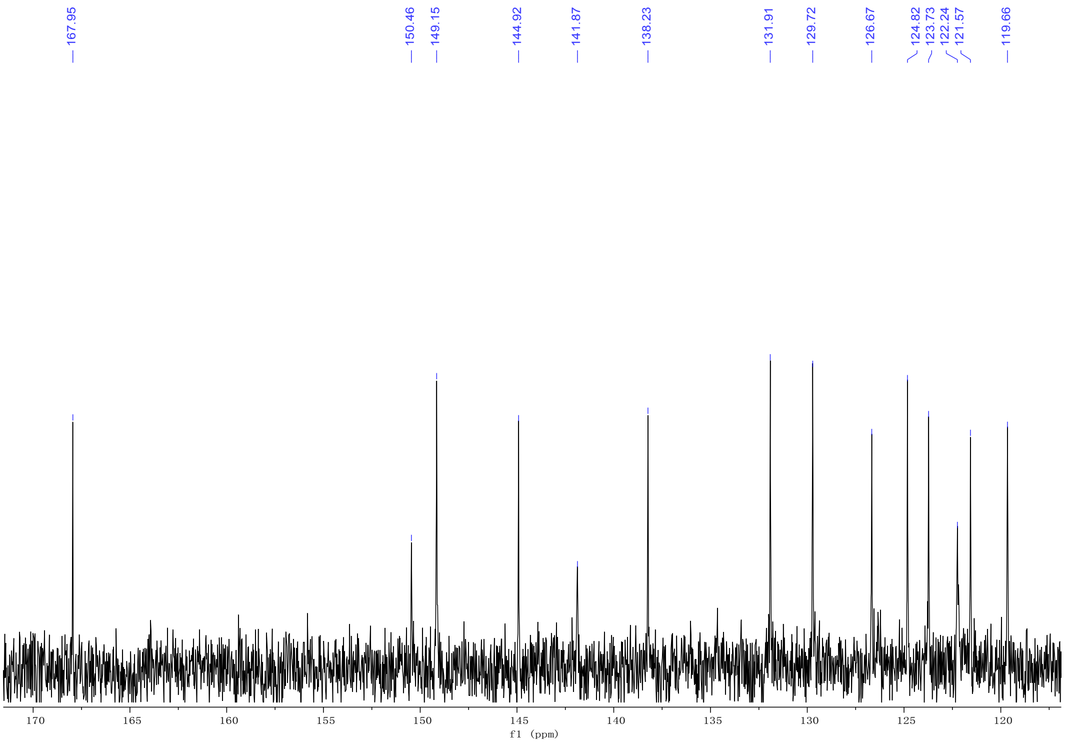

Fig. S2 ^{13}C NMR spectrum of **Ir1** in DMSO-d_6.

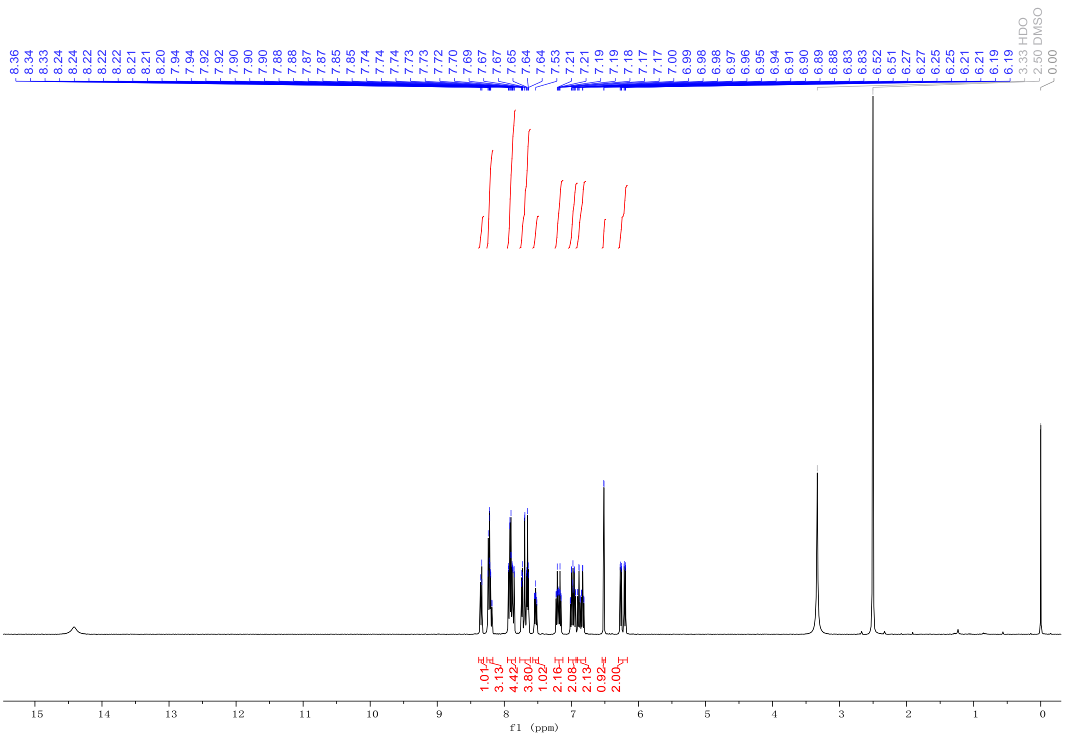

Fig. S3 ^{1}H NMR spectrum of **Ir2** in DMSO-d_6.

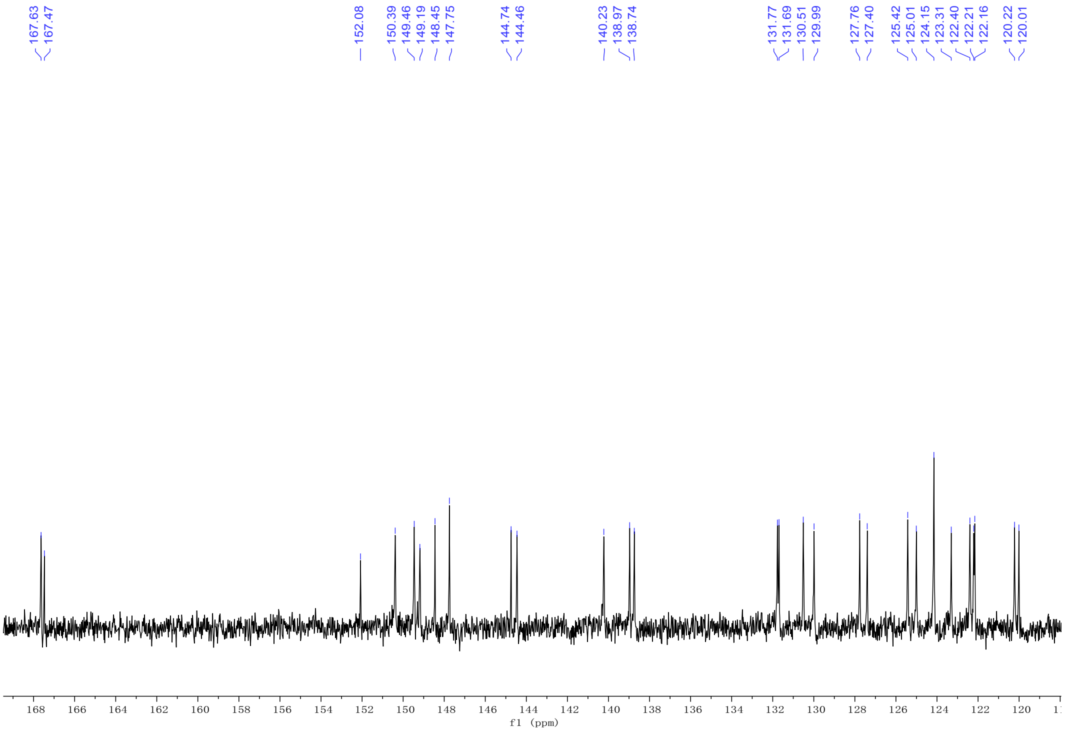

Fig. S4 ^{13}C NMR spectrum of **Ir2** in DMSO-d_6.

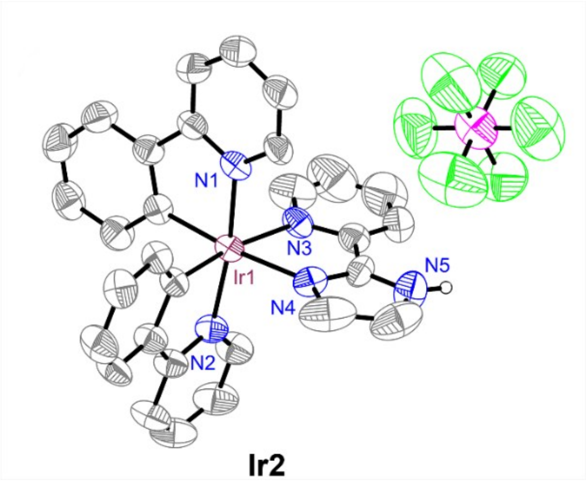

Fig. S5 X-ray structures of **Ir2** are showed in thermal ellipsoids at the 50% probability level. For the sake of clarity, most of the hydrogen atoms are omitted except for the hydrogen atoms that attach to the nitrogen atoms.

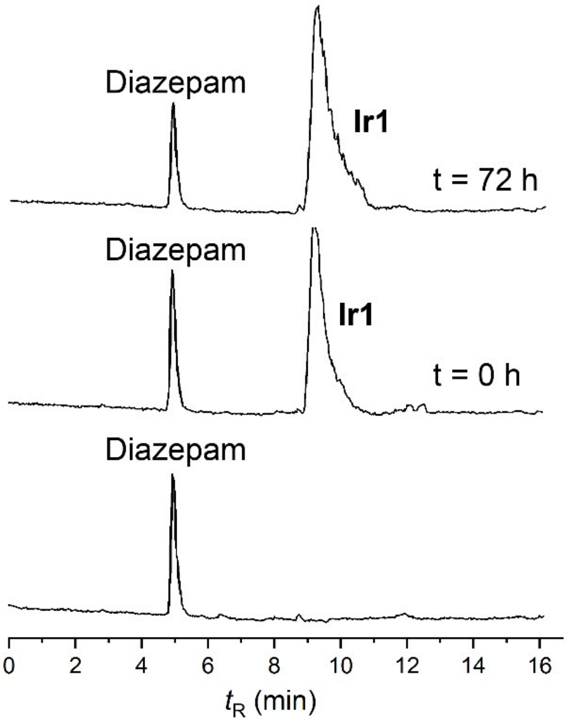

Fig. S6 HPLC-MS analysis of diazepam and **Ir1**/diazepam mixture in human blood plasma at t = 0 h and 72 h (Diazepam was used as internal standard).

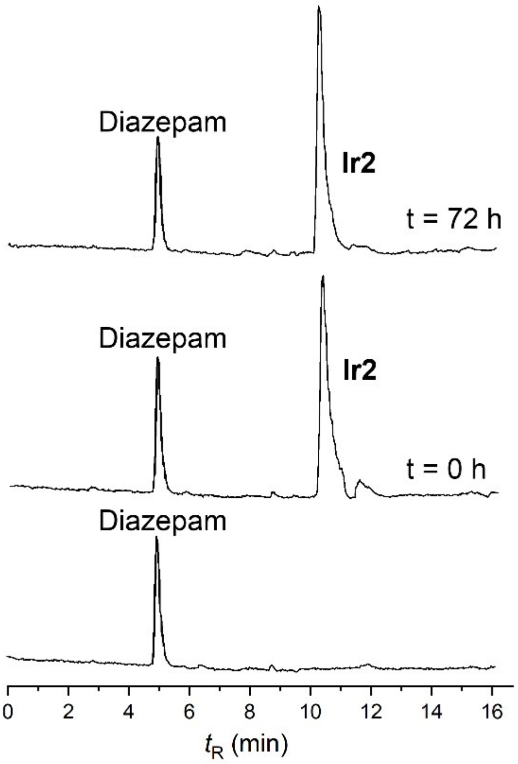

Fig. S7 HPLC-MS analysis of diazepam and **Ir2**/diazepam mixture in human blood plasma at t = 0 h and 72 h (Diazepam was used as internal standard).

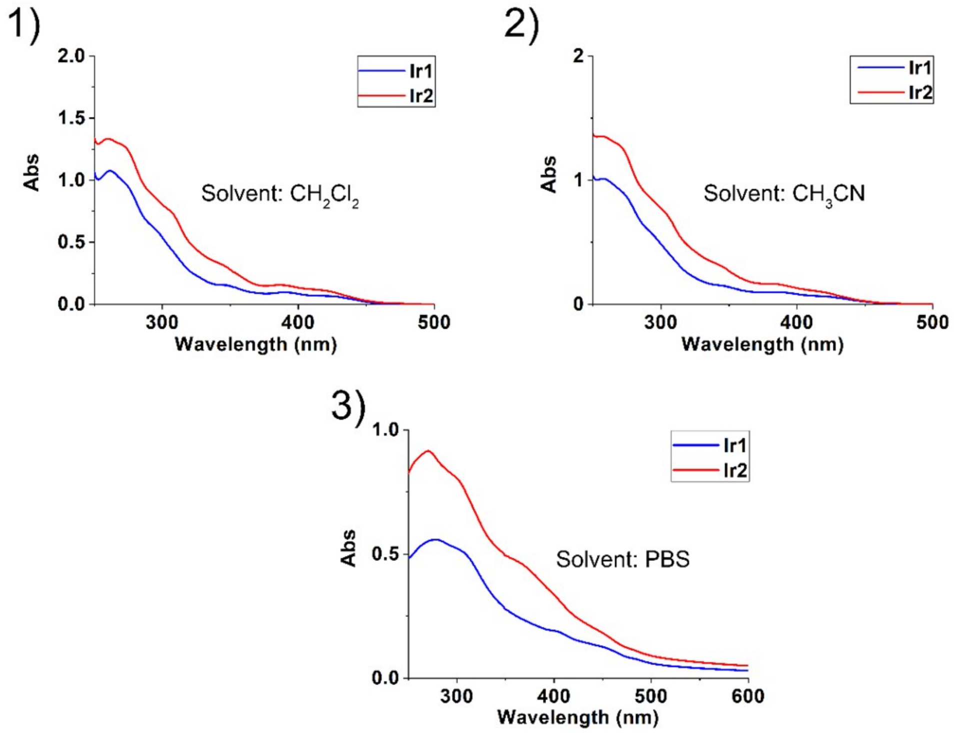

Fig. S8 UV/vis absorption spectra of complexes **Ir1** and **Ir2** (2×10^{-5} M) measured in degassed solvents ((1) CH_2Cl_2, (2) CH_3CN and (3) PBS) at 298 K.

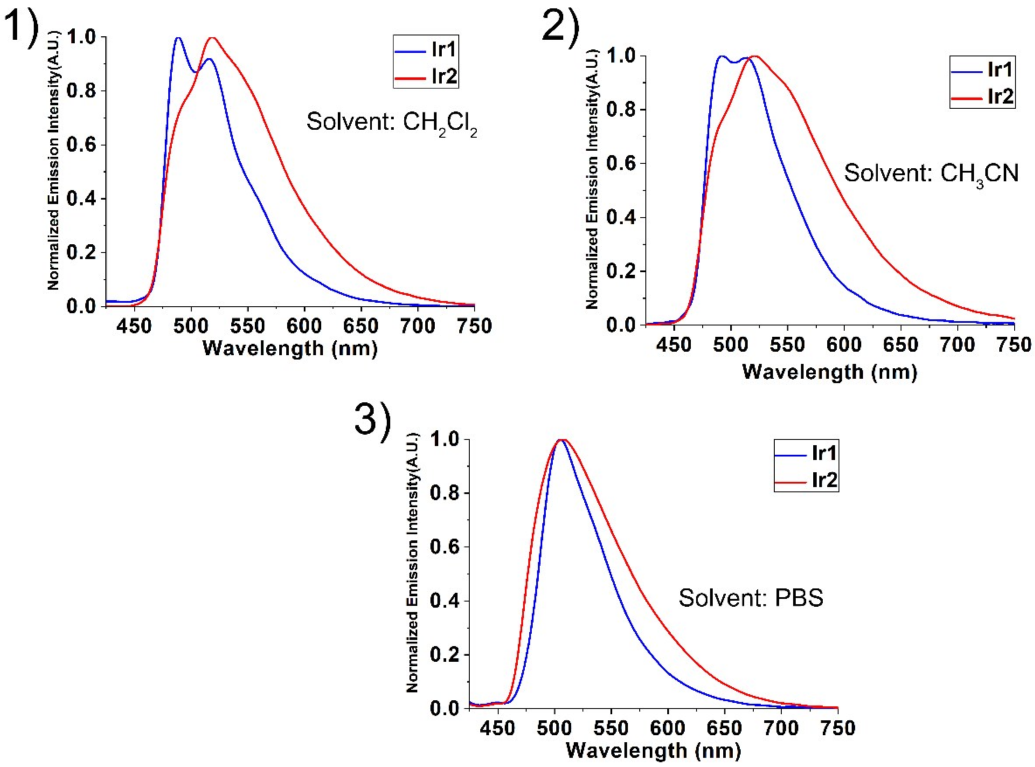

Fig. S9 Emission spectra of complexes **Ir1** and **Ir2** (2×10^{-5} M) measured in degassed solvents ((1) CH_2Cl_2, (2) CH_3CN and (3) PBS) at 298 K.

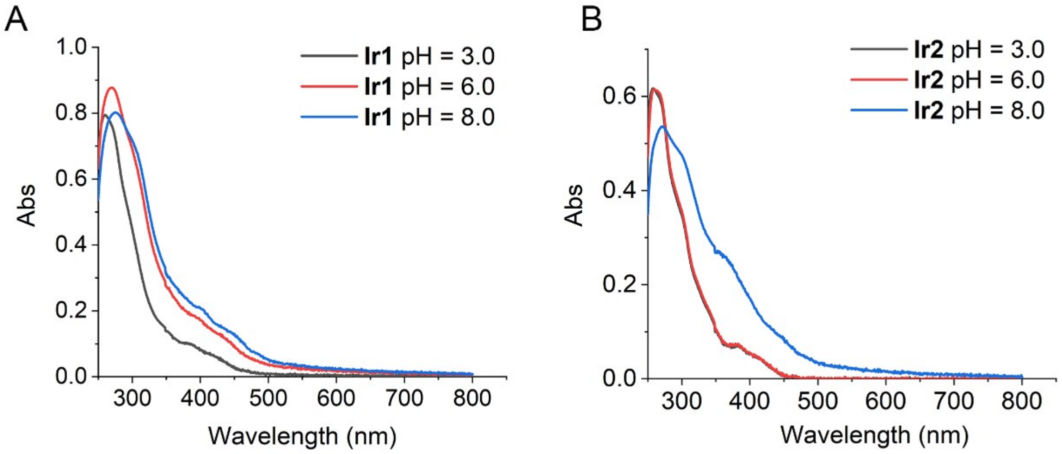

Fig. S10 UV/vis absorption spectra of complexes **Ir1** (A, 2×10^{-5} M) and **Ir2** (B, 2×10^{-5} M) measured in different pH at 298 K.

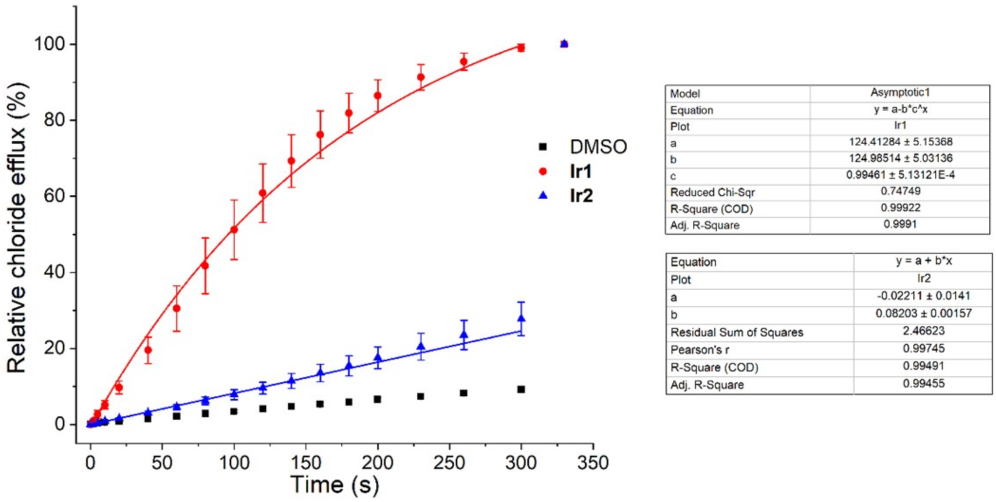

Fig. S11 Chloride/nitrate transport mediated by **Ir1** and **Ir2** (final concentration: 0.25 mol% with respect to lipid). Vesicles loaded with 500 mM NaCl with 5 mM citric-phosphate buffer (pH 7.2) were dispersed in a 500 mM $NaNO_3$ solution with 5 mM phosphate salts (pH 7.2). DMSO was used as a control. The initial rate of chloride transport (k_{ini}) was calculated through asymptotic or linear fit.

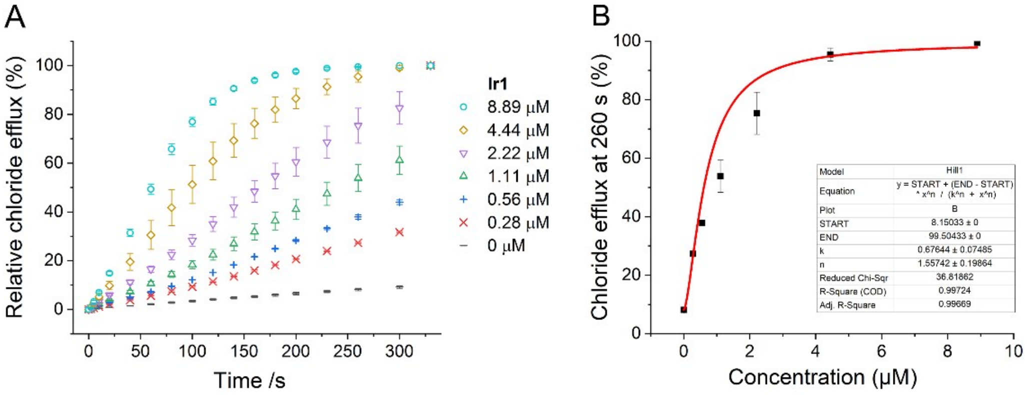

Fig. S12 (A) The relative chloride efflux of different concentrations of **Ir1**. Both of pH_{in} and pH_{out} are 7.2. (B) Hill analysis for chloride efflux mediated by **Ir1** (pH_{in} = 7.2, pH_{out} = 7.2).

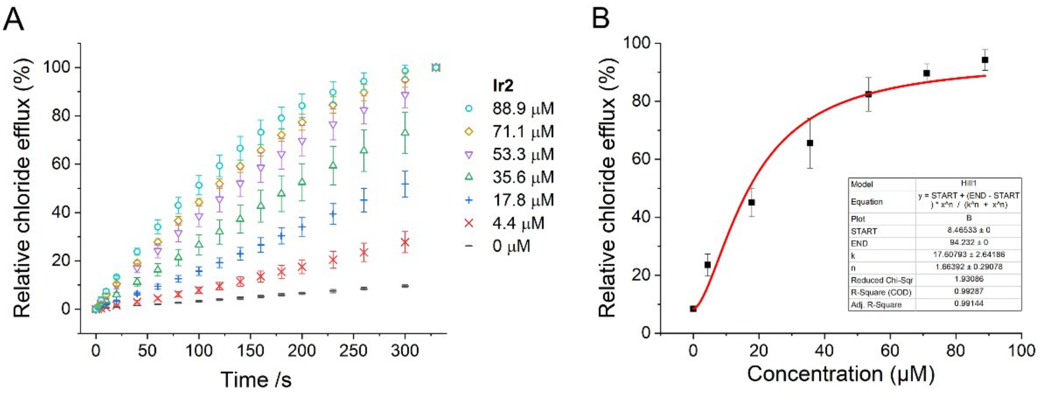

Fig. S13 (A) The relative chloride efflux of different concentrations of **Ir2**. Both of pH_{in} and pH_{out} are 7.2. (B) Hill analysis for chloride efflux mediated by **Ir2** (pH_{in} = 7.2, pH_{out} = 7.2).

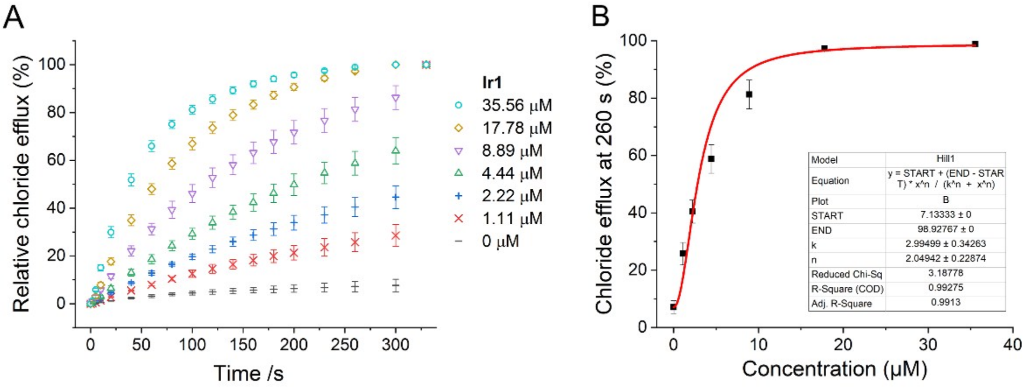

Fig. S14 (A) The relative chloride efflux of different concentrations of **Ir1**. Both of pH_{in} and pH_{out} are 6.0. (B) Hill analysis for chloride efflux mediated by **Ir1** (pH_{in} = 6.0, pH_{out} = 6.0).

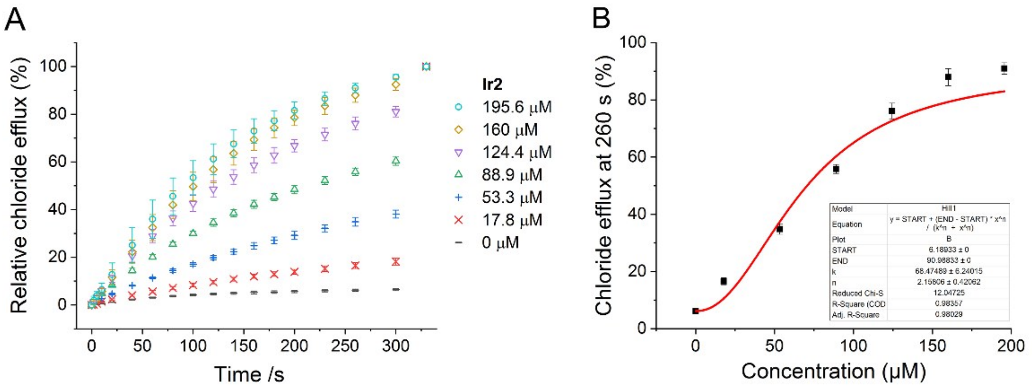

Fig. S15 (A) The relative chloride efflux of different concentrations of **Ir2**. Both of pH_{in} and pH_{out} are 6.0. (B) Hill analysis for chloride efflux mediated by **Ir2** (pH_{in} = 6.0, pH_{out} = 6.0).

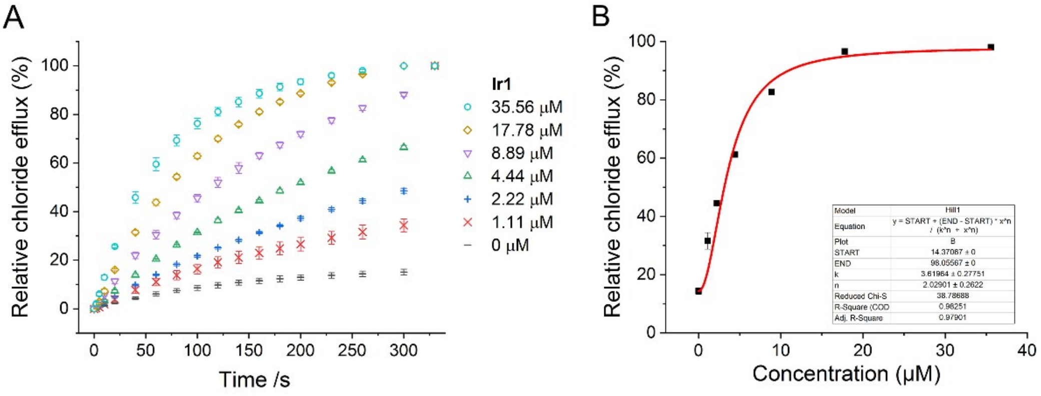

Fig. S16 (A) The relative chloride efflux of different concentrations of **Ir1**. Both of pH_{in} and pH_{out} are 5.0. (B) Hill analysis for chloride efflux mediated by **Ir1** (pH_{in} = 5.0, pH_{out} = 5.0).

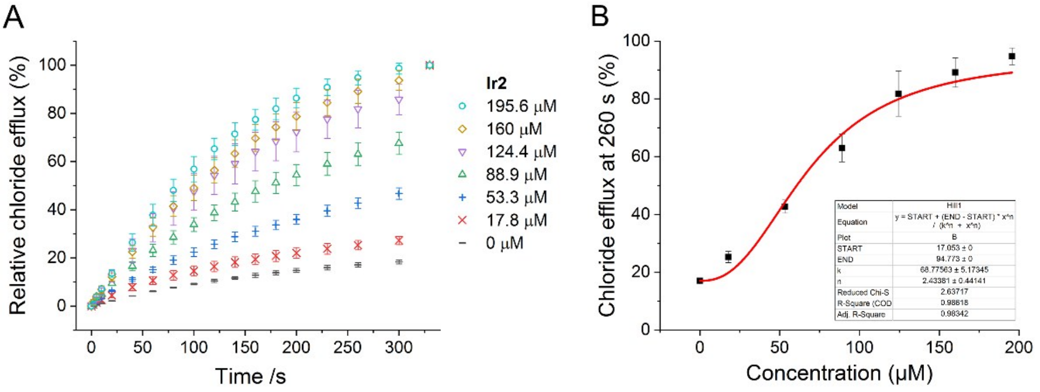

Fig. S17 (A) The relative chloride efflux of different concentrations of **Ir2**. Both of pH_{in} and pH_{out} are 5.0. (B) Hill analysis for chloride efflux mediated by **Ir2** (pH_{in} = 5.0, pH_{out} = 5.0).

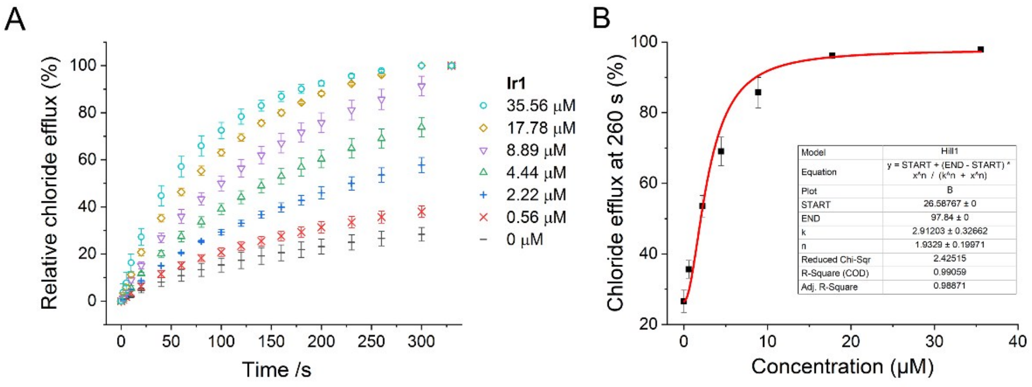

Fig. S18 (A) The relative chloride efflux of different concentrations of **Ir1**. Both of pH_{in} and pH_{out} are 4.0. (B) Hill analysis for chloride efflux mediated by **Ir1** (pH_{in} = 4.0, pH_{out} = 4.0).

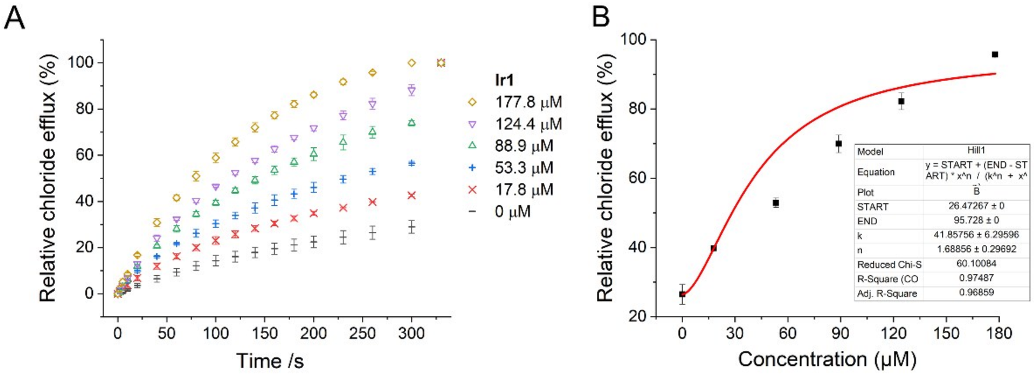

Fig. S19 (A) The relative chloride efflux of different concentrations of **Ir2**. Both of pH_{in} and pH_{out} are 4.0. (B) Hill analysis for chloride efflux mediated by **Ir2** (pH_{in} = 4.0, pH_{out} = 4.0).

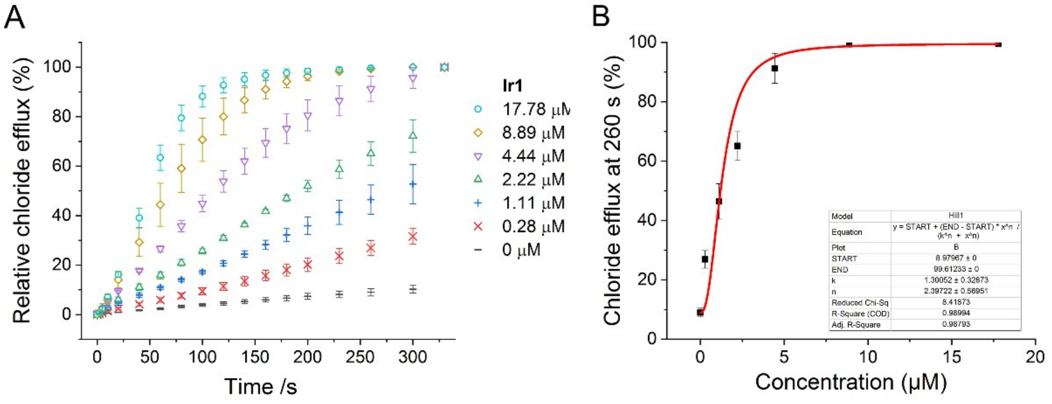

Fig. S20 (A) The relative chloride efflux of different concentrations of **Ir1** (pH_{in} = 4.0, pH_{out} = 7.2). (B) Hill analysis for chloride efflux mediated by **Ir1** (pH_{in} = 4.0, pH_{out} = 7.2).

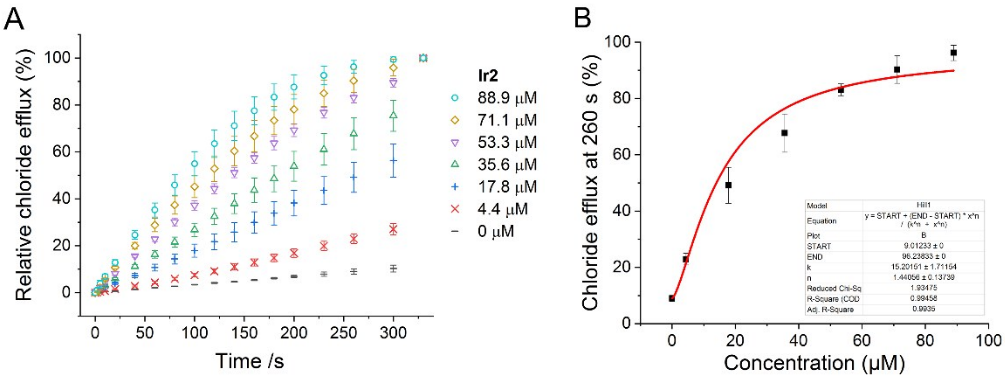

Fig. S21 (A) The relative chloride efflux of different concentrations of **Ir2** (pH_{in} = 4.0, pH_{out} = 7.2). (B) Hill analysis for chloride efflux mediated by **Ir2** (pH_{in} = 4.0, pH_{out} = 7.2).

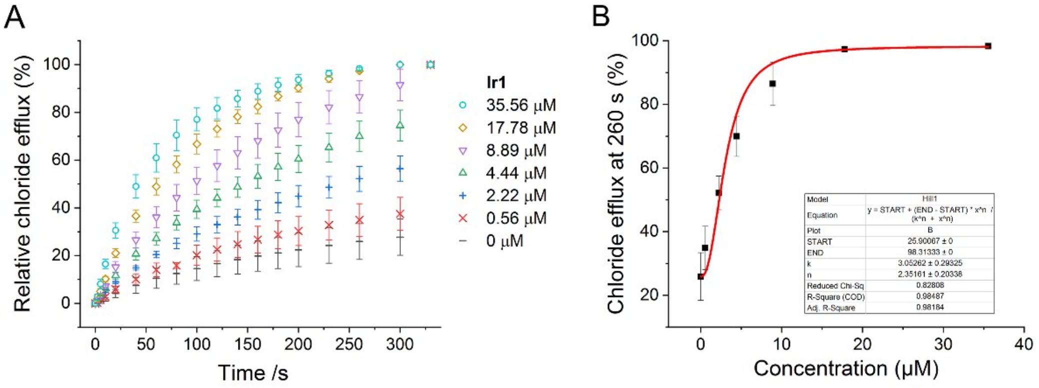

Fig. S22 (A) The relative chloride efflux of different concentrations of **Ir1** (pH_{in} = 7.2, pH_{out} = 4.0). (B) Hill analysis for chloride efflux mediated by **Ir1** (pH_{in} = 7.2, pH_{out} = 4.0).

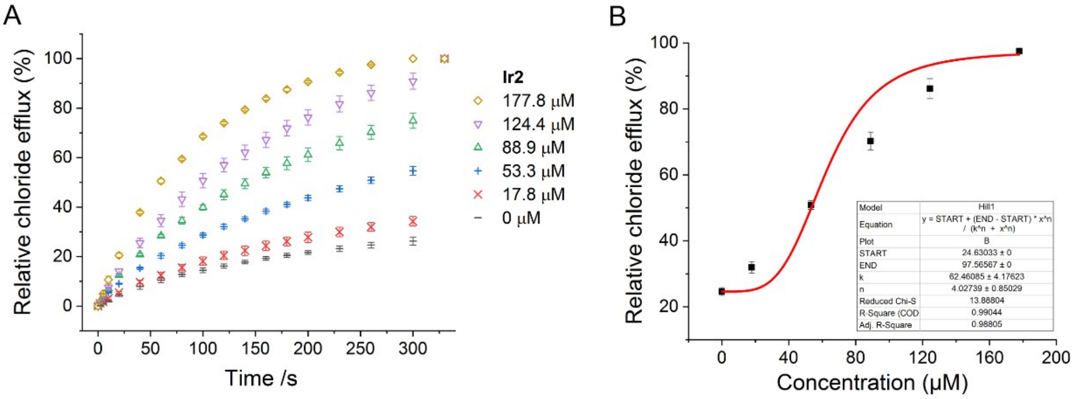

Fig. S23 (A) The relative chloride efflux of different concentrations of **Ir2** (pH_{in} = 7.2, pH_{out} = 4.0). (B) Hill analysis for chloride efflux mediated by **Ir2** (pH_{in} = 7.2, pH_{out} = 4.0).

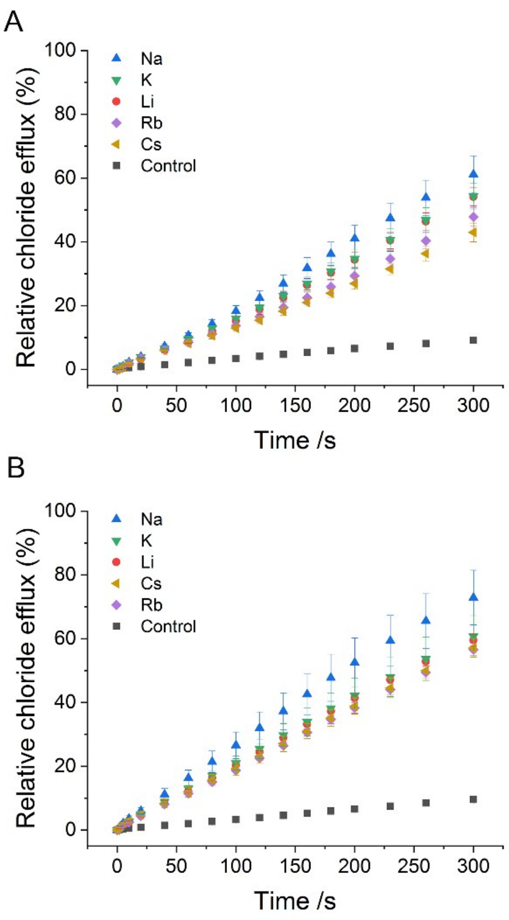

Fig. S24 The relative chloride efflux of **Ir1** (A) (1.11 μM, 0.0625 mol%) and **Ir2** (B) (35.56 μM, 2 mol%). The vesicles contained different metal chloride were suspended in $NaNO_3$ solution.

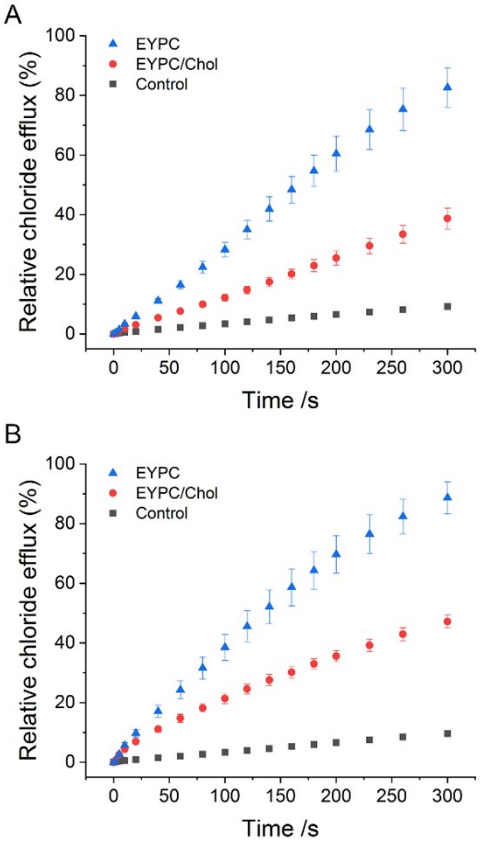

Fig. S25 The relative chloride efflux of **Ir1** (A) (2.22 μM, 0.125 mol%) and **Ir2** (B) (53.33 μM, 3 mol%). The vesicles with or without cholesterol are used for this assay.

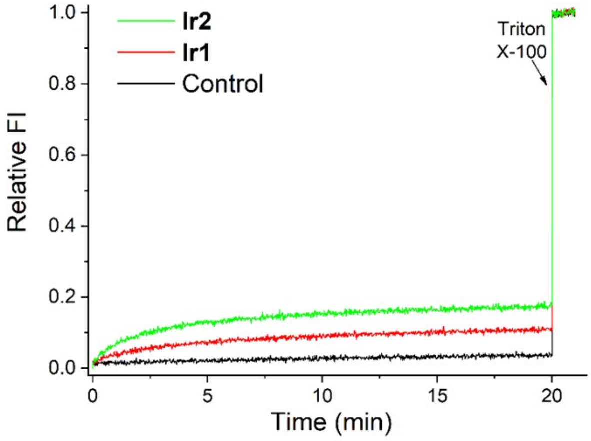

Fig. S26 Time plot of relative fluorescence intensity of calcein with **Ir1** (6.67 μM, 0.5 mol%) and **Ir2** (66.67 μM, 5 mol%).

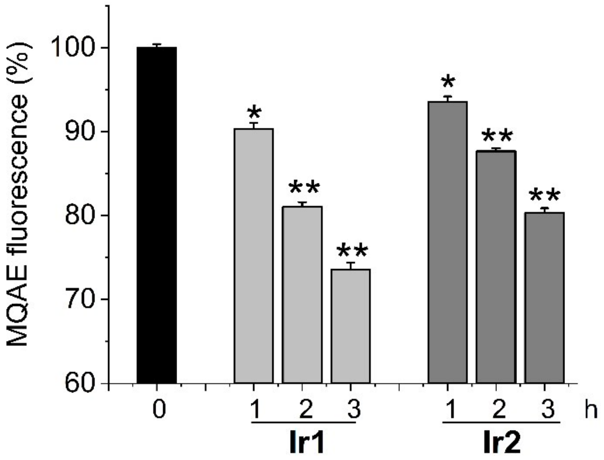

Fig. S27 A549 cells pretreated with 10 mM MQAE for 1 h were incubated with 10 μM **Ir1** or **Ir2** for the indicated times. The MQAE fluorescence was then measured to examine changes in intracellular chloride ion concentrations (mean ± S.E., n = 3). *p <0.05, **p < 0.01.

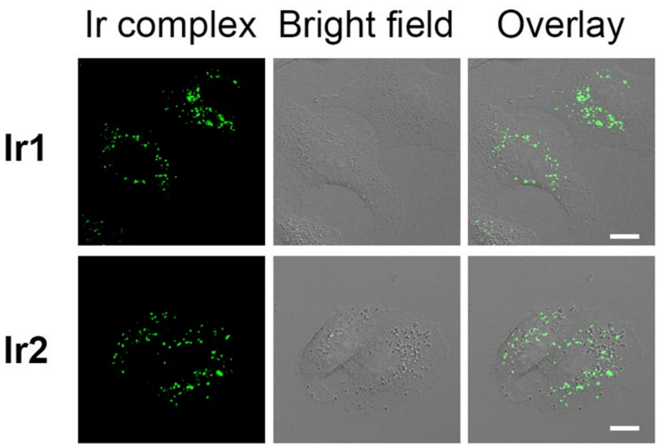

Fig. S28 Cellular uptake of **Ir1** and **Ir2** measured by confocal microscopy. A549 cells were incubated with **Ir1** and **Ir2** (4 μM) for 2 h at 37 °C. Scale bars: 10 μm. λ_{ex} = 405 nm, λ_{em} = 500 ± 20 nm.

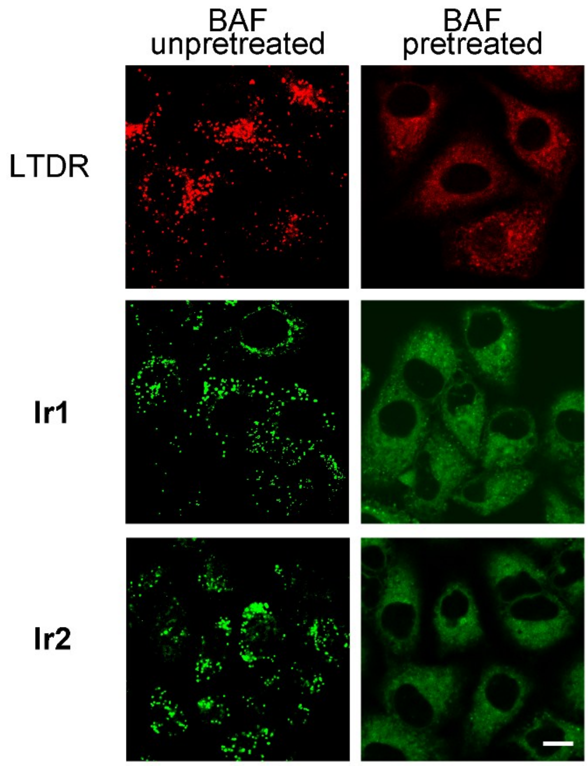

Fig. S29 Confocal microscopy images of A549 cells treated with **Ir1, Ir2** (4 μM, 2 h) or LTDR (50 nM, 0.5 h). BAF (200 nM, 1 h) was added in advance to alkalize lysosomes. Scale bar: 10 μm.

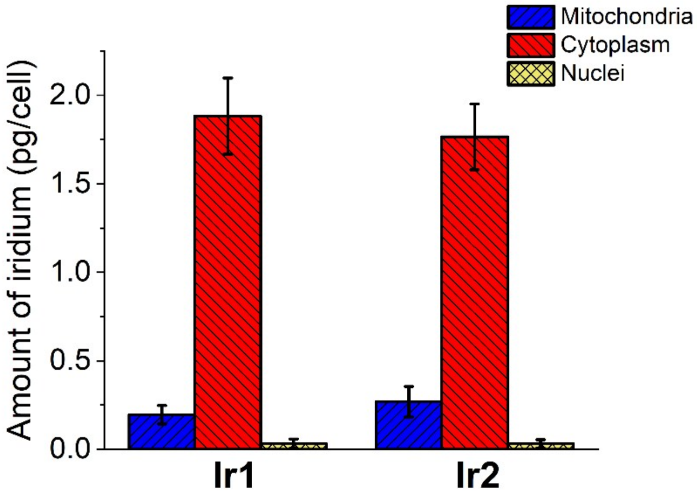

Fig. S30 Distribution of complexes **Ir1** and **Ir2** in different cellular compartments of A549 cells measured by ICP-MS. Cell were treated with Ir(III) at 10 μM for 2 h.

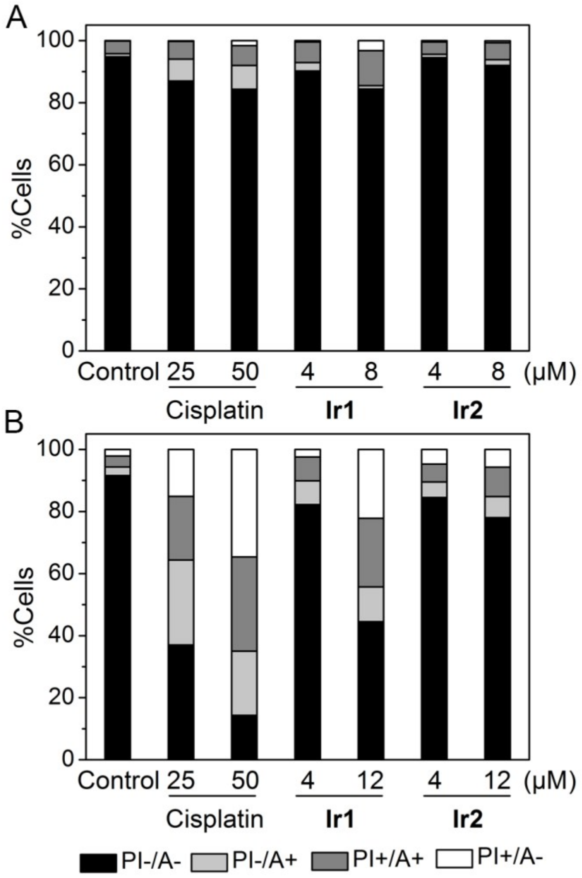

Fig. S31 Flow cytometric quantification of annexin V-FITC and PI double labeled A549 cells after treatment with cisplatin, **Ir1** and **Ir2** at the indicated concentrations for (A) 24 h or (B) 48 h.

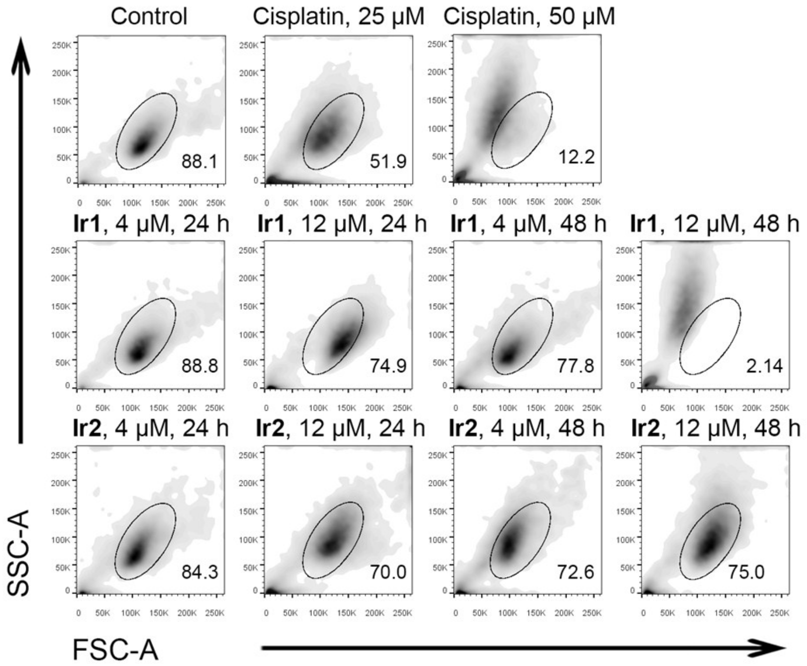

Fig. S32 Flow cytometric analysis of morphological alternation in A549 cells. Cells were incubated with **Ir1** and **Ir2** or cisplatin for 24 h or 48 h. FSC (forward scatter)/SSC (side scatter) patterns are gated for high FSC/low SSC profile (gate).

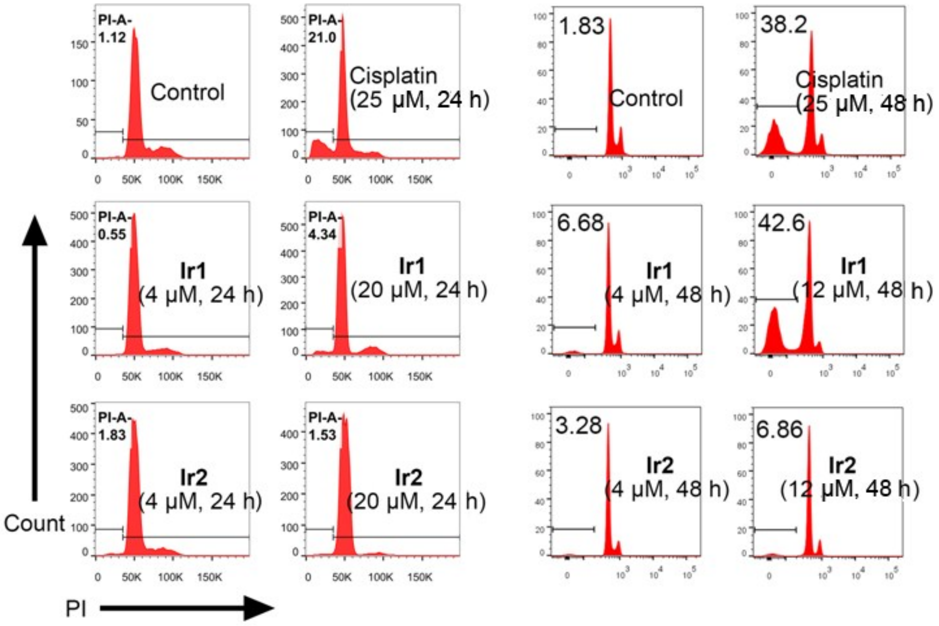

Fig. S33 A549 cells were pretreated with different concentration of **Ir1** and **Ir2** or cisplatin (Pt, 25 μM) for 24 h or 48 h prior to the addition of Triton-100 and PI. The cells were analyzed by flow cytometry.

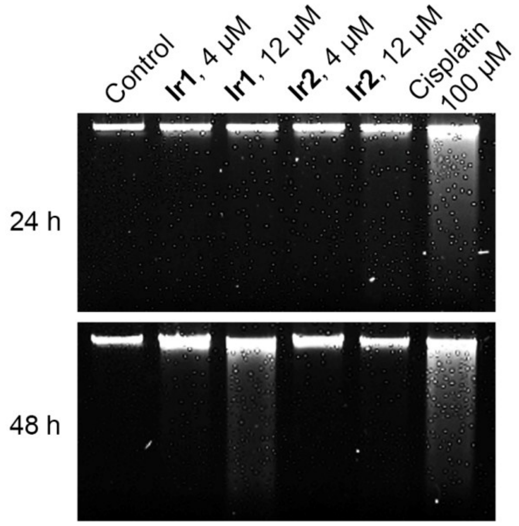

Fig. S34 Detection of DNA fragmentation in cells treated with **Ir1**, **Ir2** or cisplatin. A549 cells were treated with compounds for 24 h or 48 h. The DNA fragments were visualized by staining with nucleic acid staining solutions. Untreated cells (Control) are shown as a negative control.

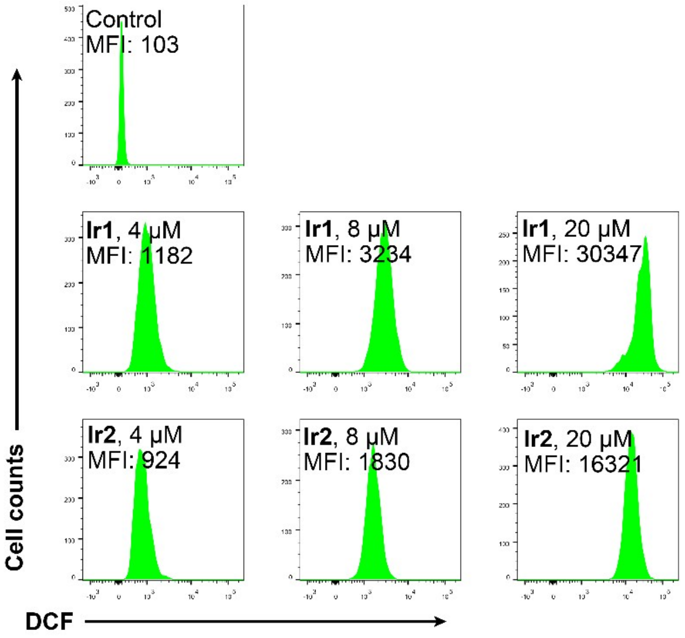

Fig. S35 Analysis of ROS levels by flow cytometry after A549 cells were treated with complexes **Ir1** and **Ir2** at the indicated concentrations for 12 h and stained with H_2DCF-DA.

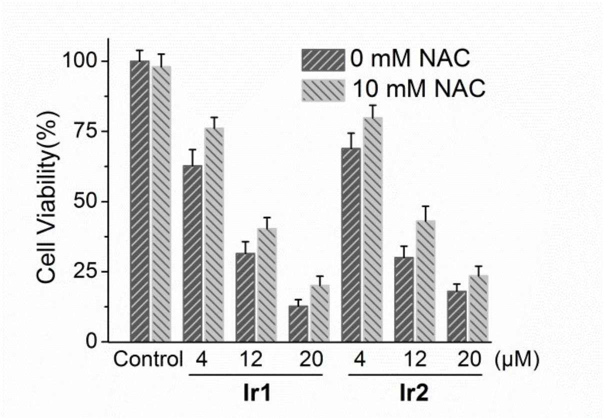

Fig. S36 Impact of NAC pretreatment on A549 cell death induced by **Ir1** and **Ir2**. A549 cells were incubated with **Ir1** or **Ir2** at the indicated concentrations for 24 h and pre-treated with NAC (10 mM) for 1 h.

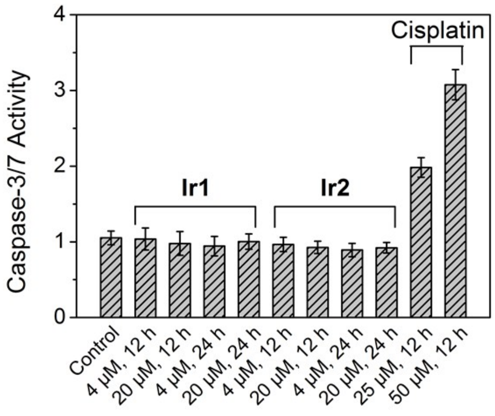

Fig. S37 Detection of caspase-3/7 activity in A549 cells treated with **Ir1**, **Ir2** or cisplatin at the indicated concentrations.

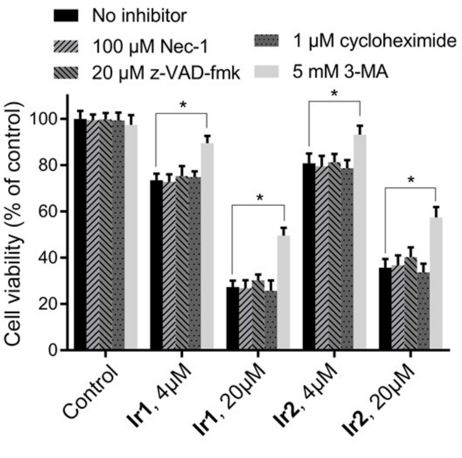

Fig. S38 A549 cells were treated with **Ir1** (4 or 20 µM) or **Ir2** (4 or 20 µM) for 24 h with or without pretreatment of Nec-1 (100 µM), z-VAD-fmk (20 µM), cycloheximide (1 µM) or 3-MA (5 mM) for 1 h. Cell viability was measured by MTT assay. Data are represented as means ± SD of three independent experiments.*$p < 0.05$.

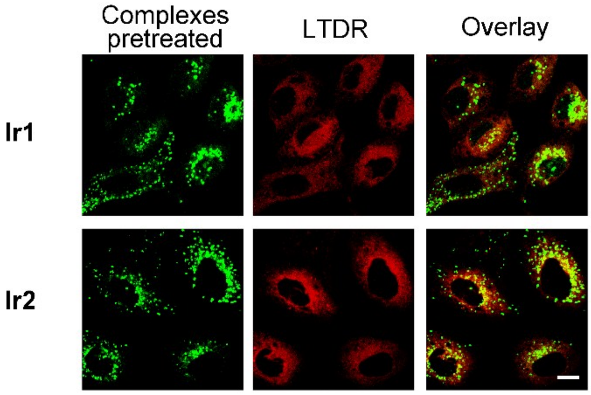

Fig. S39 Confocal microscopy images of A549 cells pretreated with **Ir1** or **Ir2** (20 µM) for 2 h and then stained with LTDR (50 nM, 0.5 h). Scale bar: 10 µm.

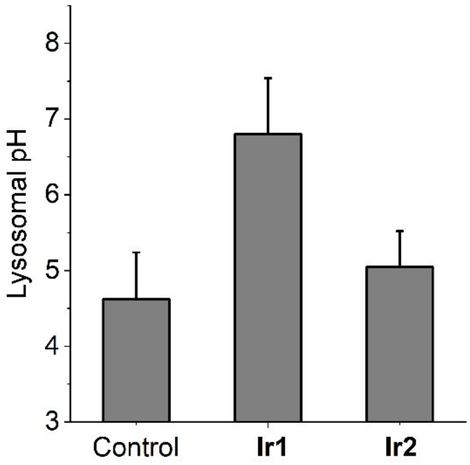

Fig. S40 The impact of **Ir1** or **Ir2** on lysosomal pH. A549 cells were pretreated with dextran labeled with fluorescein-TMR for 12 h and then incubated with 4 µM **Ir1** or **Ir2** for an additional 12 h. The pH of the lysosomes was determined by a pH titration curve (Fig. S32).

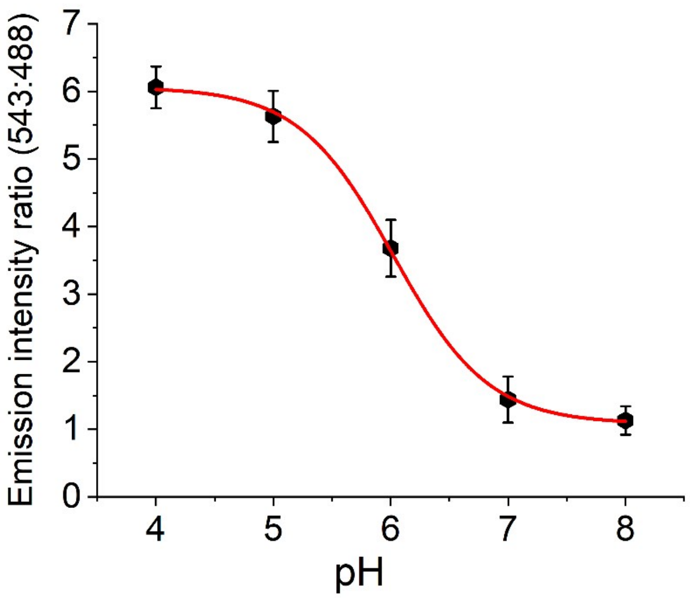

Fig. S41 The pH titration curve of fluorescein-TMR-labeled dextran. The fluorescein emission was calibrated using pH calibration buffers containing nigericin and valinomycin (mean ± s.d., n = 3).

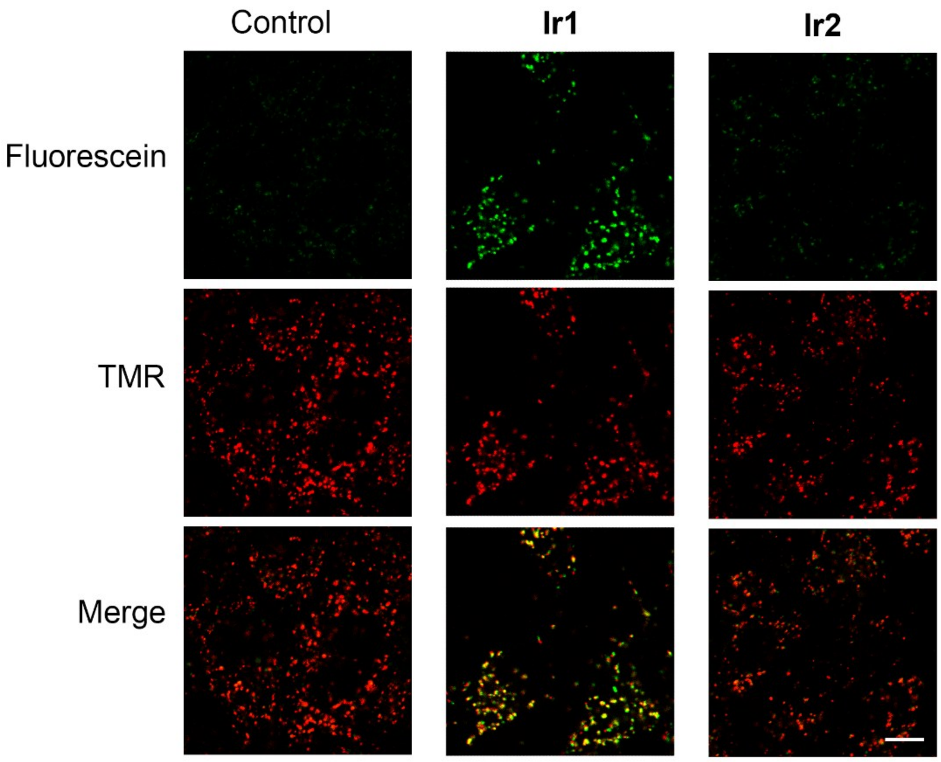

Fig. S42 Confocal images of A549 cells treated with fluorescein-TMR-labeled dextran for 12 h and then incubated with **Ir1** (4 μM) or **Ir2** (4 μM) for an additional 12 h. Scale bar = 10 μm.

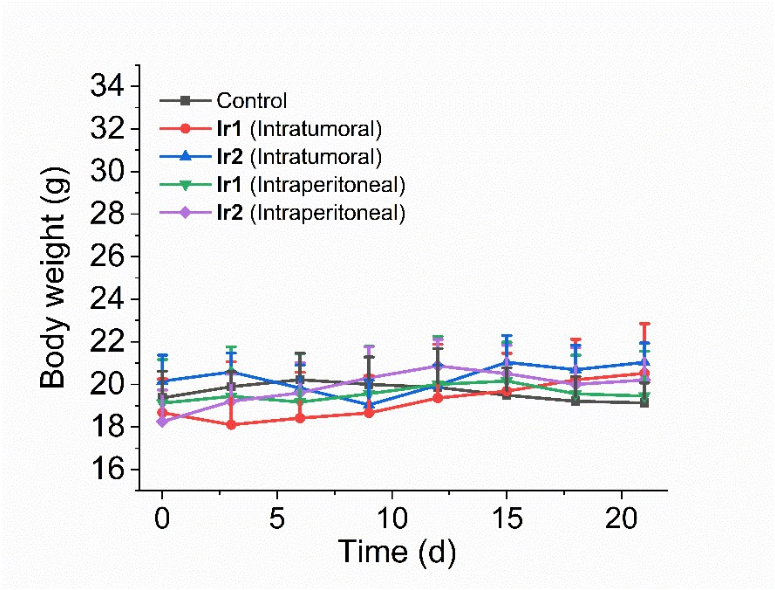

Fig. S43 Body weights of nude mice in different groups.

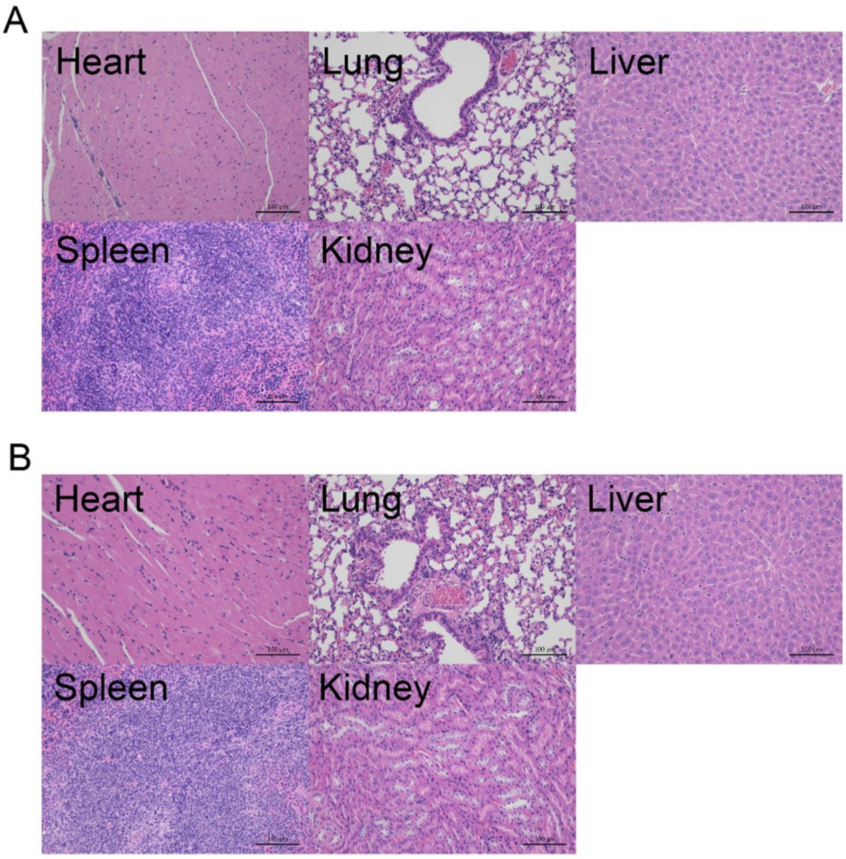

Fig. S44 H&E stained tissue images obtained from major organs of **Ir1** (A)- and **Ir2** (B)-treated mouse by intratumoral injection for in vivo toxicity evaluation. Scale bar: 100 μm.

Table S1 Crystallographic data of **Ir1** and **Ir2**

Complex	**Ir1**	**Ir2**
CDCC no.	1814116	1814117
Empirical formula	$C_{28}H_{22}F_6IrN_6P$	$C_{30}H_{23}F_6IrN_5P$
Molecular weight	779.71	790.70
Description	block	block
Temperature (K)	120(2)	292(1)
Crystal size (mm)	0.211 × 0.116 × 0.093	0.06 × 0.05 × 0.04
λ (Å)	0.71073	1.54184
Crystal system	monoclinic	orthorhombic
Space group	C2/c	Pbca
a (Å)	39.183(2)	10.9356(3)
b (Å)	9.5561(5)	15.8786(4)
c (Å)	17.8539(11)	33.0214(12)
α (°)	90	90.
β (°)	102.731(2)	90
γ (°)	90	90
Volume, Å	6520.8(7)	5733.9(3)
Z	4	8
Absorption coefficient	4.161	10.163
F(000)	2744.0	3072.0
θrange (deg)	2.543 – 28.424	4.851 – 74.727
Completeness to θ_{max}	0.995	0.990
Density (calcd) (g/cm^3)	1.440	1.832
Reflections(collected/uni	154541/8141	12602/5617
$R1^a[I > 2\sigma(I)]$	0.0313	0.0440
$wR2^a$	0.0813	0.1324
GOF^b	1.047	1.039

$$^aR1 = \sum\left\|F_0\right| - \left|F_c\right\| / \sum\left|F_0\right|, wR2 = \left\{\sum\left[w\left(F_0^2 - F_c^2\right)^2\right] / \sum\left[w\left(F_0^2\right)^2\right]\right\}^{1/2} \quad {}^bGOF = \left\{\sum\left[w\left(F_0^2 - F_c^2\right)^2 / (n-p)\right]\right\}^{1/2}$$

where n is the number of data and p is the number of parameters refined.

Table S2 Selected bond lengths (Å) and bond angles (deg) of **Ir1** and **Ir2**

Complex	**Ir1**		**Ir2**	
Bond lengths (Å)	Ir1–N1	2.042(3)	Ir1–N1	2.044(5)
	Ir1–N2	2.035(4)	Ir1–N2	2.040(5)
	Ir1–N3	2.159(4)	Ir1–N3	2.162(5)
	Ir1–N5	2.156(4)	Ir1–N4	2.132(5)
	Ir1–C11	2.003(4)	Ir1–C11	2.013(6)
	Ir1–C22	2.006(4)	Ir1–C22	1.998(6)
Bond angles (deg)	N1–Ir1–N5	96.46(13)	C11–Ir1–N1	80.5(2)
	N1–Ir1–N3	88.85(13)	C11–Ir1–N2	93.0(2)
	N5–Ir1–N3	76.04(15)	C11–Ir1–N3	99.3(2)
	C11–Ir1–N1	80.31(15)	C11–Ir1–N4	173.8(2)
	C11–Ir1–N2	95.50(15)	N2–Ir1–N3	95.8(2)
	C11–Ir1–N3	96.49(15)	N2–Ir1–N1	172.5(2)
	C11–Ir1–N5	171.99(15)	N2–Ir1–N4	91.3(2)
	C11–Ir1–C22	89.65(16)	C22–Ir1–C11	88.6(2)
	N2–Ir1–N1	173.75(13)	C22–Ir1–N3	171.4(2)
	N2–Ir1–N5	88.28(14)	C22–Ir1–N2	80.3(2)
	N2–Ir1–N3	96.26(14)	C22–Ir1–N1	95.8(2)
	C22–Ir1–N1	94.77(16)	C22–Ir1–N4	96.5(2)
	C22–Ir1–N5	97.95(16)	N1–Ir1–N3	88.9(2)
	C22–Ir1–N3	173.33(15)	N1–Ir1–N4	95.5(2)
	C22–Ir1–N2	80.51(16)	N4–Ir1–N3	75.8(2)

Table S3 Photophysical data of **Ir1** and **Ir2**[a]

Complex	Medium	Absorption λ [nm]	Emission λ [nm]	$\Phi_{em}{}^{b}$	$\tau_{av}{}^{c}$ (ns)
**Ir1**	PBS	277, 400, 449	505	0.02	132.58
	CH_3CN	258, 385, 423	492, 514	0.11	27.44
	CH_2Cl_2	261, 391, 425	489, 516	0.07	67.22
**Ir2**	PBS	271, 364, 452	507	0.12	75.96
	CH_3CN	257, 384, 418	521	0.13	30.96
	CH_2Cl_2	260, 387, 421	518	0.07	112.65

[a]All emission decays were obtained on freshly prepared samples placed in quartz cuvettes. Samples were 2×10^{-5} M in concentration. [b] Solutions of $[Ru(bpy)_3](PF_6)_2$ were used as the standard, PBS (Φ_{em} = 0.042),[10] CH_3CN (Φ_{em} = 0.062)[11] and CH_2Cl_2 (Φ_{em} = 0.059).[12] [c]Decay curves of compounds were recorded by an Edinburgh FLS 920 Spectrometer. All curves were fitted into a two exponential formula: $F(t) = A + B_1 \exp(-t/\tau_1) + B_2 \exp(-t/\tau_2) + B_3 \exp(-t/\tau_3)$ and $\tau_{ay} = \dfrac{B_1\tau_1^2 + B_2\tau_2^2 + B_3\tau_3^2}{B_1\tau_1 + B_2\tau_2 + B_3\tau_3}$.

Table S4 Anion transport activities of **Ir1** and **Ir2**

pH_{in} and pH_{out}[a]	Complex	$EC_{50,\ 260s}$ (Mean±SD) (μM)	$EC_{50,\ 260s}$ (Mean±SD) (mol %)[b]	Hill coefficient
7.2 and 7.2	**Ir1**	0.676±0.075	0.038±0.004	1.56
	**Ir2**	17.608±2.642	0.989±0.148	1.66
6.0 and 6.0	**Ir1**	2.995±0.343	0.168±0.019	2.05
	**Ir2**	68.475±6.240	3.847±0.351	2.16
5.0 and 5.0	**Ir1**	3.620±0.278	0.203±0.016	2.03
	**Ir2**	68.776±5.173	3.864±0.291	2.43
4.0 and 4.0	**Ir1**	2.912±0.327	0.164±0.018	1.93
	**Ir2**	41.858±6.296	2.352±0.354	1.69
4.0 and 7.2	**Ir1**	1.301±0.329	0.073±0.018	2.40
	**Ir2**	15.202±1.712	0.854±0.096	1.44
7.2 and 4.0	**Ir1**	3.053±0.293	0.172±0.016	2.35
	**Ir2**	62.461±4.176	3.509±0.235	4.03

[a] "pH_{in}" represents the pH in the vesicles. "pH_{out}" represents the pH outside the vesicles.

[b]The molar percentage of carrier to EYPC.

Vesicles loaded with 500 mM NaCl with 5 mM different pH citric-phosphate buffer dispersed in a 500 mM $NaNO_3$ solution with 5 mM different pH phosphate salts.

Supporting References

1. G. Stupka, L. Gremaud and A. F. Williams, *Helv. Chim. Acta*, 2005, **88**, 487-495.
2. J. S. Casas, A. Castiñeiras, Y. Parajó, A. Sánchez, Á. Sánchez-González and J. Sordo, *Polyhedron*, 2005, **24**, 1196-1202.
3. G. A. Crosby and J. N. Demas, *J. Phys. Chem.*, 1971, **75**, 991-1024.
4. R. M. Lima, M. A. D. Ferreira, T. M. d. J. Ponte, M. P. Marques, O. M. Takayanagui, H. H. Garcia, E. B. Coelho, P. S. Bonato and V. L. Lanchote, *J. Chromatogr. B*, 2009, **877**, 3083-3088.
5. G. Sheldrick, *Acta Crystallogr. A*, 2008, **64**, 112-122.
6. L. He, C. P. Tan, R. R. Ye, Y. Z. Zhao, Y. H. Liu, Q. Zhao, L. N. Ji and Z. W. Mao, *Angew. Chem., Int. Ed.*, 2014, **53**, 12137-12141.
7. W.-H. Chen and S. L. Regen, *J. Am. Chem. Soc.*, 2005, **127**, 6538-6539.
8. M. J. McKeage, S. J. Berners-Price, P. Galettis, R. J. Bowen, W. Brouwer, L. Ding, L. Zhuang and B. C. Baguley, *Cancer Chemother. Pharmacol.*, 2000, **46**, 343-350.
9. J.-J. Cao, C.-P. Tan, M.-H. Chen, N. Wu, D.-Y. Yao, X.-G. Liu, L.-N. Ji and Z.-W. Mao, *Chem. Sci.*, 2017, **8**, 631-640.
10. J. Van Houten and R. J. Watts, *J. Am. Chem. Soc.*, 1976, **98**, 4853-4858.
11. J. V. Caspar and T. J. Meyer, *J. Am. Chem. Soc.*, 1983, **105**, 5583-5590.
12. D. Pucci, A. Bellusci, A. Crispini, M. Ghedini, N. Godbert, E. I. Szerb and A. M. Talarico, *J. Mater. Chem.*, 2009, **19**, 7643-7649.
